# Efficient Electronic Tunneling Governs Transport in Conducting Polymer-Insulator Blends

**DOI:** 10.1021/jacs.2c02139

**Published:** 2022-06-06

**Authors:** Scott T. Keene, Wesley Michaels, Armantas Melianas, Tyler J. Quill, Elliot J. Fuller, Alexander Giovannitti, Iain McCulloch, A. Alec Talin, Christopher J. Tassone, Jian Qin, Alessandro Troisi, Alberto Salleo

**Affiliations:** † Department of Materials Science and Engineering, Stanford University, Stanford, California 94305, United States; ‡ Department of Chemical Engineering, Stanford University, Stanford, California 94305, United States; § Sandia National Laboratories, Livermore, California 94551, United States; ∥ Department of Chemistry, University of Oxford, Oxford OX1 3TA, U.K.; ⊥ SLAC National Accelerator Laboratory, Stanford Synchrotron Radiation Light Source, Menlo Park, California 94025, United States; # Department of Chemistry, University of Liverpool, Liverpool L69 3BX, U.K.

## Abstract

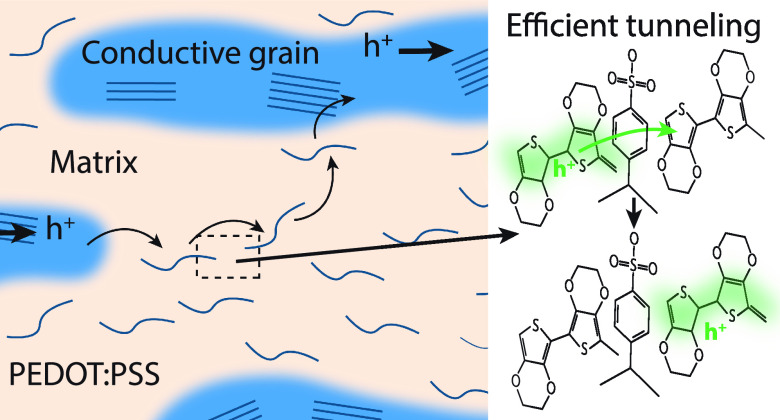

Electronic transport models for conducting polymers (CPs) and blends focus on the arrangement of conjugated chains, while the contributions of the nominally insulating components to transport are largely ignored. In this work, an archetypal CP blend is used to demonstrate that the chemical structure of the non-conductive component has a substantial effect on charge carrier mobility. Upon diluting a CP with excess insulator, blends with as high as 97.4 wt % insulator can display carrier mobilities comparable to some pure CPs such as polyaniline and low regioregularity P3HT. In this work, we develop a single, multiscale transport model based on the microstructure of the CP blends, which describes the transport properties for all dilutions tested. The results show that the high carrier mobility of primarily insulator blends results from the inclusion of aromatic rings, which facilitate long-range tunneling (up to *ca.* 3 nm) between isolated CP chains. This tunneling mechanism calls into question the current paradigm used to design CPs, where the solubilizing or ionically conducting component is considered electronically inert. Indeed, optimizing the participation of the nominally insulating component in electronic transport may lead to enhanced electronic mobility and overall better performance in CPs.

## Introduction

All conducting polymer (CP) films are effectively mixtures of at least one conducting and one non-conducting component. In the design of CPs, the processibility of rigid conjugated polymer backbones are tuned through side-chain engineering^1−3^ or by blending with non-CPs^4−6^ to obtain composite properties. Indeed, relatively minor changes to the side chain or polymer blend composition can result in substantial improvements in their performance.

Microstructure plays a crucial role in determining the electronic and mixed conducting properties that make CPs technologically relevant. Often phase segregation results in ordered, semiconductor-rich domains, which are mixed with disordered regions with poor transport properties.^7−9^ CPs are thus designed to either minimize inter-grain distance or to provide pathways such as tie-chains to bridge the conductive regions. Indeed, the size, shape, distribution, and interconnectivity of ordered semiconductor grains controlled *via* molecular weight,^8^ degree of crystallinity,^2^ solvent modification,^10,11^ annealing^12,13^ and post-treatments^14^ have all been demonstrated to affect the electronic conductivities by improving the inter-grain percolation. However, by treating transport as a percolative process, which focuses solely on the morphology and connectivity of aggregated phases, minority transport within disordered or insulator-rich phases is ignored.^15−17^

**Figure 1:**
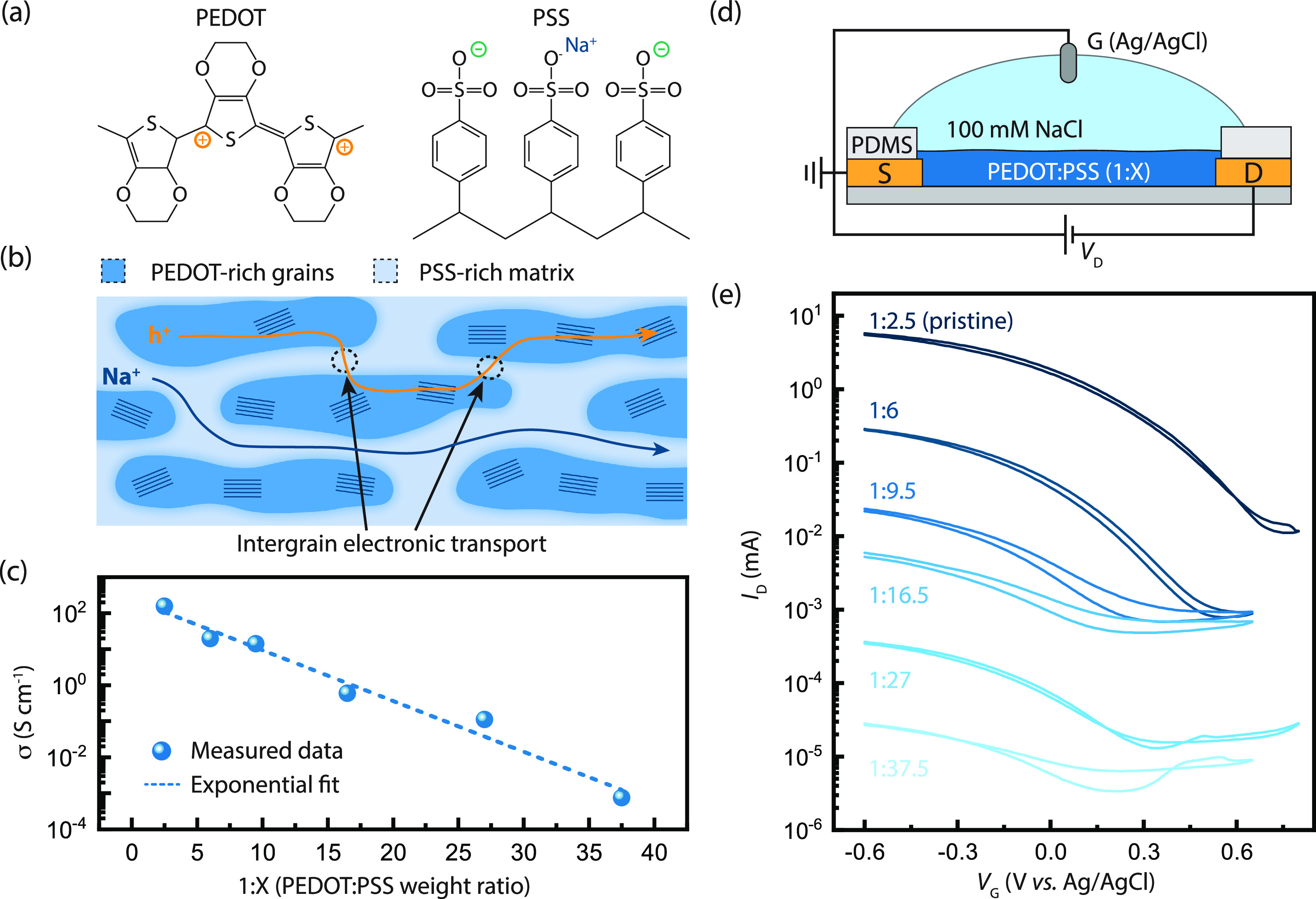
Conductivity scaling of increasingly diluted PEDOT:PSS blends. (a) Chemical structures of poly(ethylene dioxythiophene) (PEDOT) and poly(styrene sulfonate) (PSS). (b) Schematic showing electronic (orange) and ionic (dark blue) transport through a representative PEDOT:PSS microstructure. (c) Conductivity of PEDOT:PSS blends with increasing PSS concentration measured at room temperature with a 4-point probe technique. (d) Schematic diagram of an organic electrochemical transistor (OECT) test structure. (e) Transfer characteristics of OECTs made using increasingly diluted PEDOT:PSS blends.

In many CP-insulator blends, a low conductivity phase surrounds conductive grains, and thus, electronic transport is often limited by the transit through this region.^8^ This type of microstructure is notably present in poly(ethylene dioxythiophene):poly(styrene sulfonate) (PEDOT:PSS, Figure 1a), which consists of PEDOT-rich grains embedded in a presumed insulating PSS-rich matrix (Figure 1b).^18^ PEDOT:PSS, however, displays a remarkably high conductivity (*G* ∼ 4300 S cm^–1^)^19^ and electronic mobility (μ ∼ 11.7 cm^2^ V^–1^ s^–1^)^20^ that approaches that of pure PEDOT films doped by other means (*G* ∼ 6300 S cm^–1^, μ ∼ 18.5 cm^2^ V^–1^ s^–1^).^21^ This high mobility is surprising given that typical PEDOT:PSS blends consist of *ca.* 71 wt % of the insulating polymer PSS (*ca.* 10^–8^ S cm^–1^).^22^ Thus, understanding the mechanism for such efficient inter-grain transport in PEDOT:PSS would aid in the design of new materials that displays improved inter-grain transport.

Blending and side-chain engineering have also recently emerged as a tool to give CPs the ability to transport ions, constituting organic mixed ionic–electronic conductors (OMIECs).^23^ OMIECs are attractive because they enable a wide range of emerging applications in the bioelectronics and energy storage space. For these applications, the ionic conductivity of the material, mediated by regions swollen with the electrolyte, is crucial for enabling bulk electrochemical (de)doping modulated by an applied electric field.^23−25^ However, there is an inherent trade-off in the design of OMIECs: as the ion conducting pathway is improved, it disrupts percolation through the electronic conducting pathway.^11^ Thus, there is a need for materials designed to balance ionic and electronic transport^26,27^ to optimize the performance of organic electrochemical devices such as transistors^24^ or neuromorphic devices.^28^ In particular, improving electronic transport in the ion conducting phase is essential for efficient redox switching since the electronic charges in this region are displaced first during electrochemical (de)doping.^11,29,30^

In this work, we change the composition of CP-insulator blends to investigate the electronic transport in the insulator-rich, ionically conductive phase. The results show that electronic transport through non-conducting components is not inherently inefficient. That is, electronic transport in insulator-rich regions can proceed rather efficiently *via* a hitherto overlooked tunneling mechanism, the efficiency of which depends sensitively on the chemical makeup of the insulating component. Using a multiscale transport model based on molecular dynamics (MD) simulated microstructures, we show that a single interchain tunneling mechanism can capture the charge transport properties for CP blends containing anywhere from 37 to 2.6 wt % conductor. Furthermore, we make the broader point that in mixed phase CPs and blends, electronic transport cannot be simplified to percolation through a composite material made of a conducting phase and a purely insulating phase. Instead, transport in the assumed-insulating phase is non-negligible and can be dramatically enhanced through the introduction of aromatic functional groups, which facilitate long-range tunneling (*ca.* 2.1–2.9 nm) between isolated CP chains. The implications of our work extend to systems of interest for thermoelectrics^31^ or artificial synapses,^32^ where blending is used to fine tune the electronic conductivity and charge density of the resulting materials, as well as for electrochemical devices^24^ utilizing mixed ionic and electronic conduction which require efficient electronic transport in ionically conductive phases.^25^

## Results and Discussion

Using PEDOT:PSS as a model system, we dilute the CP PEDOT from *ca.* 71 wt % PSS (1:2.5 weight ratio PEDOT to PSS, pristine) down to *ca.* 97.4 wt % PSS in the blend (1:37.5 weight ratio PEDOT to PSS) and observe a monoexponential decrease in the conductance in solid-state films spanning nearly 6 orders of magnitude (Figure 1c). The resulting spin-cast thin films show uniform surface morphologies, indicating that homogeneous dispersions are achieved following dilution (Figure S1). Notably, electrochemical modulation of the channel conductance observed for PEDOT:PSS OECTs operating in an aqueous electrolyte (Figure 1d) is preserved for all weight ratios tested (Figure 1e). This preservation of electrochemical (de)doping indicates that the transport processes studied here apply both dry CP films and electrochemical devices. We note that further dilutions have been demonstrated in working electrochemical devices^32^ but were omitted from this study due to the limited sensitivity of our electrical measurement equipment and the resolution of the fabrication process.

### Electrical Characterization

**Figure 2:**
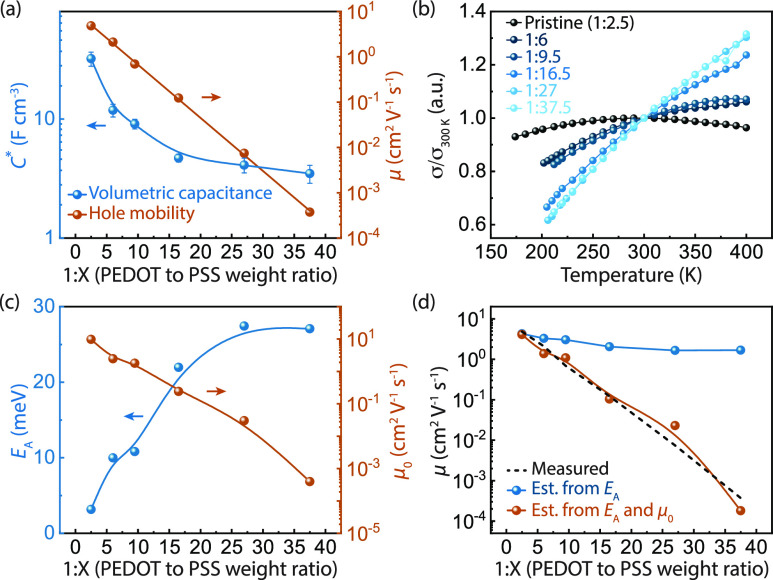
Characterization of charge transport in diluted CP blends. (a) Volumetric capacitance (orange) and mobility (blue) scaling for increasingly diluted PEDOT:PSS blends. (b) Temperature-dependent van der Pauw conductivity measurements for diluted PEDOT:PSS blends. (c) Activation energy *E*_A_ (blue) and mobility pre-factor μ_0_ (orange) fits for the temperature dependent conductivity measurements based on a thermally activated hopping transport model (see Figure S5). (d) Comparison of the measured mobility (dashed line) with the estimated room-temperature mobility using the activated hopping model varying only *E*_A_ while holding μ_0_ constant (blue) and including both *E*_A_ and μ_0_ (orange).

To understand the conductivity scaling of diluted PEDOT:PSS blends, we separated charge carrier density (inferred from the volumetric capacitance, C*, as described in Figure S1) from charge carrier mobility, μ (Figure 2a). We find that the volumetric capacitance measured using electrochemical impedance spectroscopy roughly scales with the weight fraction of PEDOT in the PEDOT:PSS solution (Figure S2). Interestingly, the hole mobility μ in the PEDOT:PSS blends (measured with OECTs using a previously reported current injection technique,^33^ see Figure S3) decays exponentially with increasing dilution, similar to the conductivity. This result indicates that the decreased conductivity is predominantly caused by a degradation of charge transport, while the decrease in the carrier concentration plays a minor role. To ensure that the μ values measured with OECTs are relevant to dry films, we estimated the mobility of the dry films using the conductivity, as shown in Figure 1c, and estimated charge carrier density from Figure S2 and found agreement between the independent calculations (Figure S4). Notably, the slope of the mobility scaling curve is constant over several orders of magnitude, indicating that a single transport mechanism should describe all sampled dilutions.

We characterized the temperature-dependent transport properties in PEDOT:PSS blends using four-point probe conductivity measurements (Figure 2b) in a van der Pauw geometry (Figure S5). The activation energy (*E*_A_) and mobility pre-factor (μ_0_) (Figure 2c) are extracted by fitting the low-temperature regimes of the curves (Figure S5) to a simple, commonly used Arrhenius form
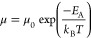
1where *k*_B_ is the Boltzmann constant and *T* is the temperature in Kelvin. While *E*_A_ does depend on dilution, all *E*_A_ values are near or below the thermal energy *k*_B_*T*, indicating that the activation barrier for charge transport does not play a strong role in the observed mobility scaling. Indeed, if we compare the measured mobility (Figure 2d, dashed) to the mobility computed assuming a constant pre-factor μ_0_ (Figure 2d, blue), it is clear that the change in *E*_A_ alone fails to capture the transport behavior of diluted PEDOT:PSS blends. Conversely, if the variation of μ_0_ is included (Figure 2d, orange), the mobilities calculated based on the Arrhenius fits match the experimentally measured mobility values.

Using a variable-range hopping (VRH) formalism^34^ which describes the hopping rate *v* as a function of the hop range, *R*, as a combination of the energetic and spatial contributions is given as follows
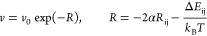
2where *v*_0_ is the hop attempt frequency, *R*_ij_ is the hopping distance, α is the inverse localization radius, and Δ*E*_ij_ is the difference in energy between the two sites. Thus, we expect that the decrease in mobility with dilution is related to increased hopping distance, *R*_ij_, which is temperature independent, rather than increased energetic disorder.

### Structural Characterization

**Figure 3:**
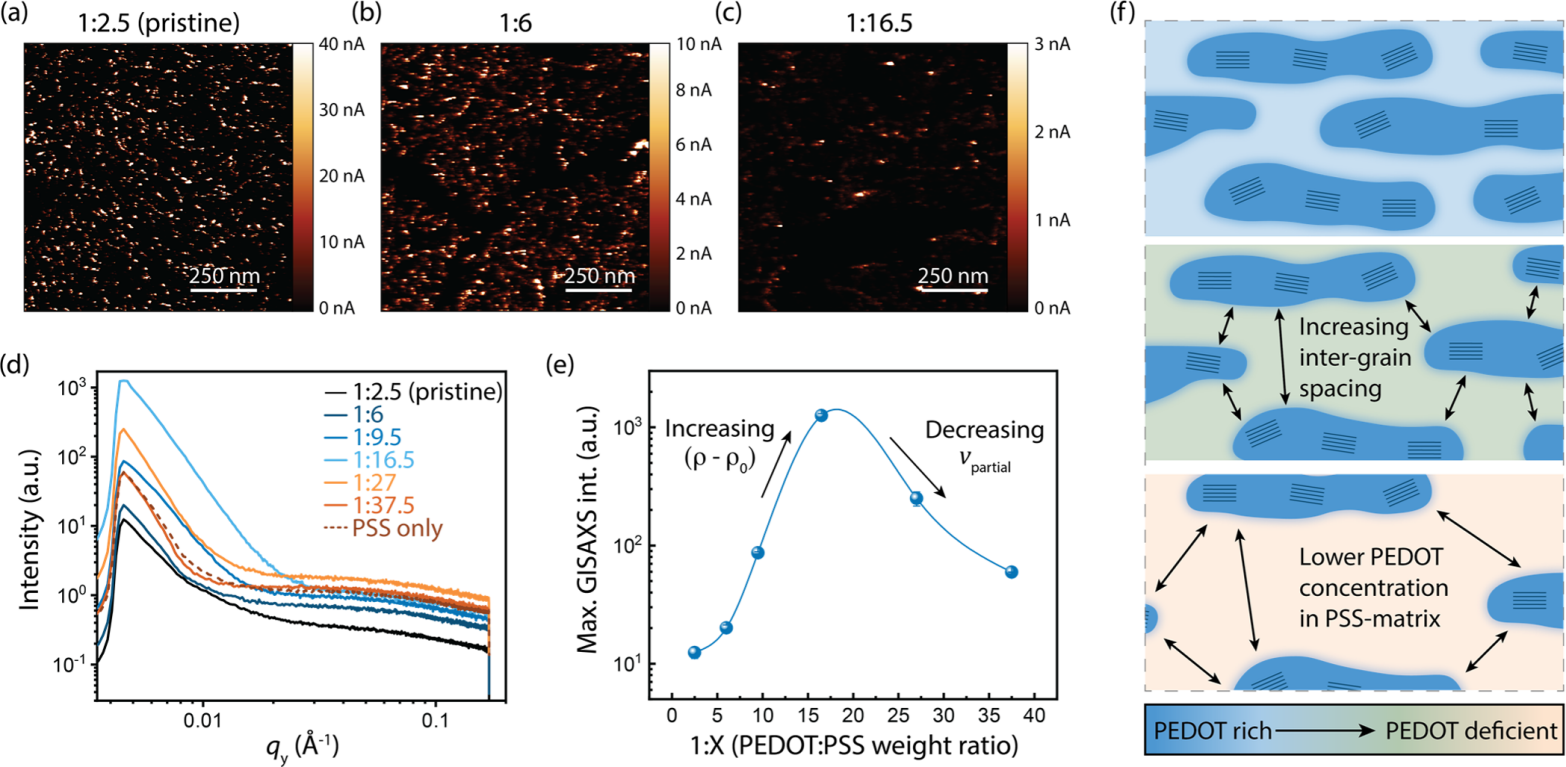
Structural characterization of diluted PEDOT:PSS blends. c-AFM images for (a) 1:2.5 (pristine), (b) 1:6, and (c) 1:16.5 PEDOT:PSS blends. (d) GISAXS and (e) peak intensity for PEDOT:PSS blends. (f) Schematic demonstrating the structural effect of dilution with excess PSS; the spacing between PEDOT-rich grains increases while the PEDOT concentration in the PSS-rich matrix decreases.

To identify the role of microstructure in the mobility scaling of PEDOT:PSS, we used conductive atomic force microscopy (c-AFM) to identify conductive regions in PEDOT:PSS films (Figure 3a–c). As expected from previous studies,^11,12,18^ in pristine PEDOT:PSS, we observe conductive grains dispersed in a low-conductivity matrix, which are attributed to the PEDOT-rich and PSS-rich regions, respectively (Figure 3a). As the blend is diluted with increasing PSS, the conductive grains remain roughly the same size and shape while the inter-grain spacing increases (Figure 3b,c). This result is consistent with the presence of insoluble PEDOT-rich aggregates within the PEDOT:PSS dispersion, which do not dissolve as PSS is added to solution. Thus, as the total number of aggregates in the dispersion decreases, the grains at the surface of the film become spaced farther apart.

To characterize the bulk microstructure of PEDOT:PSS films, we used both grazing-incidence small-angle X-ray scattering (GISAXS) (Figure 3d) and grazing-incidence wide-angle X-ray scattering (GIWAXS). GIWAXS characterization (Figure S6) confirms that PEDOT-rich aggregates are present in the film at high dilution ratios, verifying the phase separation even in the diluted samples. From GISAXS, we observe that the maximum scattering intensity first increases with decreasing PEDOT content up to the 1:16.5 dilution, then decreases (Figure 3e). The magnitude of the thickness normalized GISAXS scattering intensity *I*_0_ for PEDOT:PSS thin films can be described by
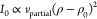
3where *v*_partial_ is the volume fraction of scatterers, and (ρ – ρ_0_) is the difference in electron density between the scattering particles and the surrounding matrix. The scattering in PEDOT:PSS is due to the difference in density (and therefore electronic density) between PEDOT-rich and PSS-rich phases^35^ (1 g cm^–3^ for PEDOT:PSS and 0.8 g cm^–3^ for PSS). Previous findings using resonant soft X-ray scattering suggest that the concentrations of PEDOT in the PSS-rich region and in the aggregates are comparable (40–45 wt % in the aggregates *vs* 30–37 wt % in the matrix),^11^ which explains the low GISAXS peak intensity for the pristine PEDOT:PSS blend. With increased dilution, we expect the volume fraction of PEDOT-rich regions to decrease (Figure 3a–c), which should cause a decrease in scattering intensity. However, the scattering intensity increases with dilution and thus must be due to the increasing difference in density between the PEDOT-rich and PSS-rich phases. Because the PEDOT aggregates are insoluble in water and likely have a fixed PEDOT:PSS weight ratio, we attribute the increase in scattering intensity to a decrease in the PEDOT concentration within the PSS-rich matrix. Eventually at higher dilutions, the lowered volume fraction of PEDOT-rich particles causes the total scattering intensity to decrease (Figure 3e).

### Multiscale Transport Model

By combining the electrical and structural characterization, we can narrow down the possible mechanisms responsible for the observed conductivity scaling and infer the conduction mechanism in undiluted blends (for a more detailed discussion, see Section S1). The electrical results indicate that the PSS-rich phase is not completely insulating, and therefore, we rule out direct tunneling across the large distances (estimated as 25–60 nm, see Section S2) separating PEDOT grains in highly dilute samples. This large interparticle spacing is also longer than the expected length of individual PEDOT chains (*ca.* 3–7 nm),^36^ so we exclude the role of single PEDOT tie chains bridging conductive regions. The exponential decay of the conductivity with dilution contradicts the assumption of a constant resistivity within the PSS-rich phase, thus conflicting with previous generalized effective media models.^15^ Finally, the constant scaling over a wide range of concentrations without a critical threshold for conduction is inconsistent with percolation models^16,17^

**Figure 4:**
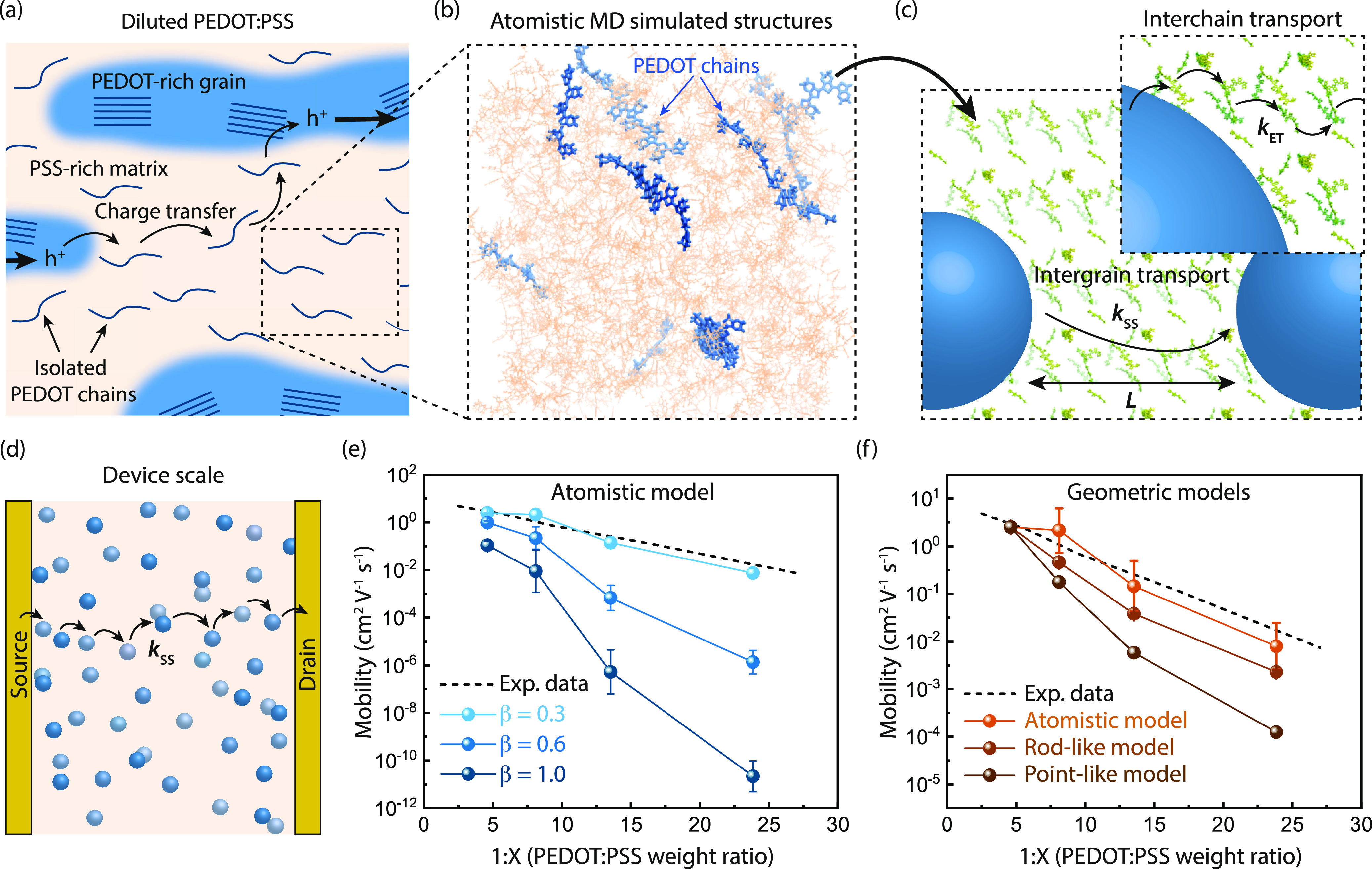
Computational transport model in PEDOT:PSS films. (a) Schematic of diluted PEDOT:PSS films, where PEDOT chains in PSS regions facilitate charge transport (arrows) between PEDOT-rich regions (dark blue ellipsoids). (b) Simulated arrangement of PEDOT chains in the PSS-rich matrix, which are tessellated to generate the (c) molecular and meso scale charge transport (CT) model. At the molecular scale, holes depart from a PEDOT-rich region (blue sphere, bottom left) and tunnel between PEDOT chains (yellow-green molecules) through the PSS-rich matrix. *k*_ET_ is defined in eq S1. At the mesoscale, holes move between PEDOT-rich regions at an effective rate, *k*_ss_(*L*), calculated with eqs S4–S6 where the grain-to-grain distance *L* is between 25 and 60 nm (see Section S2). (d) At the device scale, holes move through the film by traveling between randomly dispersed PEDOT-rich grains. (e) Mobility curves derived from MD simulated structures with varying tunneling attenuation coefficients, β, compared to the experimentally measured mobilities of diluted PEDOT:PSS (dashed line). (f) Mobility curves from experiment (dashed line) and transport models using various geometric estimates of PEDOT chains with β = 0.3 Å^–1^. Colored traces in (e,f) are shifted vertically for clarity.

Instead, the data are only consistent with transport between PEDOT-rich grains being limited through the PSS-rich matrix, where the rate of inter-grain transport must depend on the weight fraction of PSS. The simplest explanation is that charges transport through the PSS-rich matrix *via* impurity conduction^37,38^ mediated by redox-active sites (*e.g.*, PEDOT chains) originating from the PEDOT:PSS blend. The GISAXS results indicate that PEDOT is present within the PSS-rich phase, and the concentration of PEDOT in the PSS-rich phase decreases as the blend is diluted (Figure 3f). Additionally, the low activation energy of transport (Figure 2c) suggests delocalized charges on the charge transfer (CT) sites, consistent with holes along PEDOT chains. Finally, because of the glassy character of PEDOT:PSS films, we neglect charge transport *via* diffusion of redox mediators and infer that charges must tunnel from one dispersed PEDOT site to the next in order to transit through the PSS-rich matrix (Figure 4a).

To describe transport through the PSS-rich phase, we developed a model based on previous results in steady-state CT networks.^39^ We used atomistic MD simulations^40,41^ to generate representative configurations of PEDOT molecules in the PSS-rich matrix (Figure 4b, details in Section S3). From the simulations, we sample the nearest-neighbor distances and compute the tunneling rate between chains. By tessellating the simulated structures, we extrapolate to find the transport rates between PEDOT-rich grains (Figure 4c and Section S4) (see eq S1 in the Experimental Methods section). Finally, the intergranular transport rates are used to compute the device scale mobilities based on a modeled distribution of spherical PEDOT-rich grains within the PSS-rich matrix (Figure 4d). Upon dilution, both the average tunneling distance and inter-grain distance increase. The computed charge carrier mobilities (Figure 4e, blue traces) reproduce the exponential scaling in the experimental data (Figure 4e, dashed line), with the tunneling attenuation coefficient β only altering the slope of the curve. Furthermore, we note that the combined effect of increasing the inter-grain spacing and interchain tunneling distance results in the qualitative mobility scaling behavior and is thus unaffected by the precise nature of the dilution assumptions made (Table S1 and Figure S7).

Finite size effects of PEDOT chains affect transport at high PEDOT concentrations in the PSS-rich matrix. When the geometries of PEDOT chains in the model are simplified and treated as point-like particles (Figure 4f and Section S5), the mobility decays sharply with dilution compared to structures generated using MD. By estimating PEDOT chains as rodlike particles (Figure S8), the estimated mobilities approach the experimental results. However, the best fit results from the MD generated structures. This is a result of the macromolecular nature of PEDOT, expressed through its molecular length, which reduces the tunneling distance between PEDOT oligomers, thereby enhancing the electronic transport. This predicted geometric effect matches previous experimental results, showing a sharp increase in mobility with degree of polymerization for short-chain lengths.^8^ As the PEDOT concentration decreases, all geometries follow similar mobility scaling, demonstrating that as the tunneling distance between individual PEDOT chains increases (Figure S9, Section S6), their size and orientation have a diminishing effect on transport.

To match the experimentally measured mobilities, the attenuation coefficient for tunneling through PSS is estimated as β ∼ 0.3 Å^–1^ (Figure 4e). This β value is representative of tunneling attenuation factors of organic molecules with aromatic moieties and is substantially lower than values for alkanes (which are typically used for side chains), which have β values of *ca.* 0.9 Å^–1^.^42^ This low tunneling attenuation coefficient indicates a factor of 3× improvement in interchain hopping rates compared to tunneling through alkane-rich phases. However, because charges undergo several interchain hops during conduction, exchanging PSS for a poor tunneling efficiency matrix results in a several orders of magnitude decrease in mobility. Thus, we attribute the relatively high mobilities (*ca.* 4 × 10^–4^ cm^2^ V^–1^ s^–1^) in very dilute (97.4 wt % insulator) PEDOT:PSS blends to the aromatic moieties contained on PSS chains.

### Role of Aromatic Moieties

**Figure 5:**
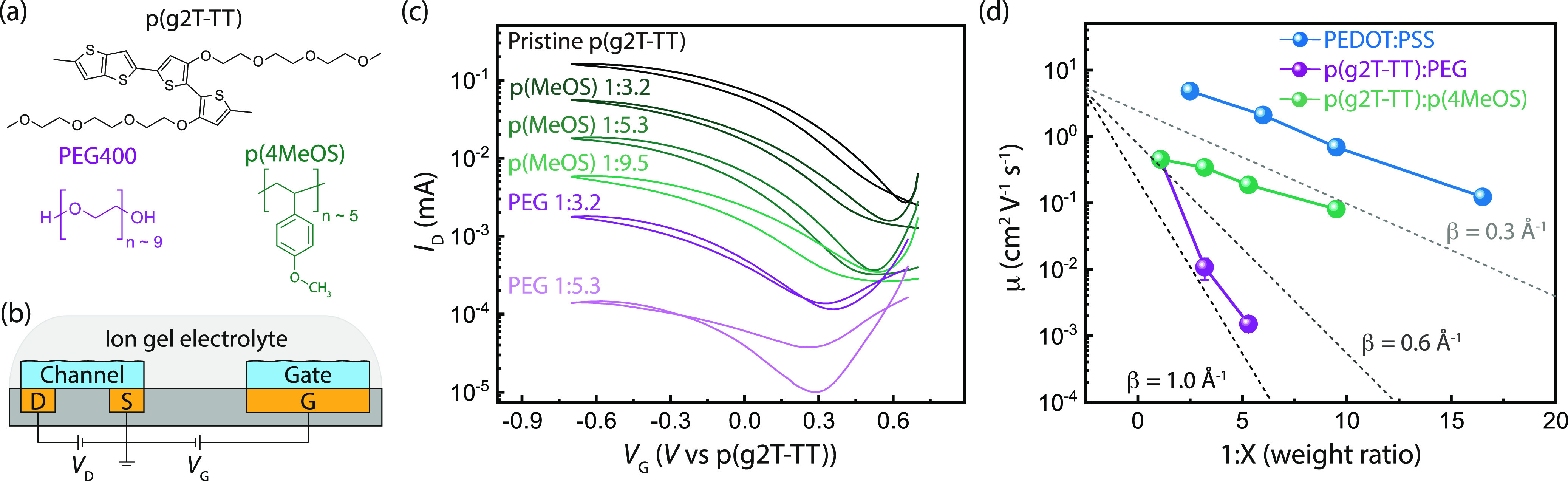
Conductivity scaling for p(g2T-TT):PEG mixed conducting blends. (a) Chemical structures for the semiconducting polymer p(g2T-TT) and blending polymers PEG and p(4MeOS). (b) OECT device test structure using an ion gel electrolyte. (c) OECT transfer characteristics for the diluted p(g2T-TT) blends. (d) Comparison of the mobility scaling PEDOT:PSS (blue), p(g2T-TT):PEG (purple), and p(g2T-TT):p(4MeOS) (green) diluted blends. For reference, the slopes corresponding to tunneling coefficients of β = 0.3, 0.6, and 1.0 Å^–1^ are plotted (gray dashed lines). We note that the weight of the pegylated side chains of p(g2T-TT) was considered as a part of the non-conductive weight fraction.

In an attempt to confirm the contribution of the aromatic groups of PSS to efficient charge transport, we considered diluting PEDOT:PSS with a non-aromatic insulator and ion conductor (polyethylene glycol, PEG), which should result in a degraded mobility. However, since PEDOT and PSS chains are bound by electrostatic interactions,^43^ and the initial PEDOT:PSS solution always has more PSS by weight than PEDOT, the isolated PEDOT chains will be locally surrounded by PSS, making a true control experiment, one where PEG forms a homogeneous matrix around PEDOT molecules, impossible. As a result, we devised a complementary strategy using an alternative, recently developed CP, poly(2-(3,3′-bis(2-(2-(2-methoxyethoxy)ethoxy)ethoxy)-[2,2′-bithiophen]-5-yl)thieno[3,2-*b*]thiophene) (p(g2T-TT))^44^ to compare transport through a matrix consisting of either a PEG (matching the chemistry as the side chain) or a PSS analogue, poly(4-methoxystyrene) [p(4MeOS)] (Figure 5a). According to our hypothesis, p(g2T-TT):p(4MeOS) blends should display enhanced mobilities compared to p(g2T-TT):PEG blends because of the improved tunneling efficiency in the ion conducting phase. Since p(g2T-TT) is an intrinsic semiconductor and is electronically insulating, we study the mobility scaling using OECTs to inject the electronic charge carriers *via* electrochemical doping. Additionally, instead of a water-based electrolyte, which would dissolve PEG, the p(g2T-TT) OECTs utilized a solid-state ion-gel electrolyte consisting of a poly(vinylidene fluoride-*co*-hexafluoropropylene) matrix with the ionic liquid, 1-ethyl-3-methylimidazolium bis(trifluoromethyl sulfonyl)imide dissolved within it (Figure 5b).

The transfer characteristics for the series of p(g2T-TT) blends were similar to those observed for PEDOT:PSS blends (Figure 5c). Blending p(g2T-TT) with PEG resulted in a decrease in mobility by 2 orders of magnitude from 0.45 cm^2^ V^–1^ s^–1^ to 1.5 × 10^–3^ cm^2^ V^–1^ s^–2^ for a relatively low dilution factor (1:5.3 by weight) (Figure 5d). However, p(g2T-TT):p(4MeOS) blends showed a only minor decreases in mobility (*ca.* 2.4× decrease) for the same weight fraction of CP (Figure 5d). Thus, by exchanging PEG for p(4MeOS), the mobility of the blend is enhanced by a factor of 32 and 124 for dilution factors of 3.2 and 5.3, respectively. Comparing the experimental curves to the estimated slopes for different tunneling attenuation coefficients (Figure 5d, gray dashed lines), we find that the mobility scaling for p(g2T-TT):PEG is comparable to a β value of *ca.* 1 Å^–1^ (as expected for non-aromatic molecules) while both PEDOT:PSS and p(g2T-TT):p(4MeOS) follow β ∼ 0.3 Å^–1^. The sharper decay of mobility observed for p(g2T-TT):PEG blends indicates that electronic transport through a PEG-based matrix is much less efficient than for blends with a polystyrene-based polymer, confirming that the presence of aromatic rings in the insulating component enhances the charge transport by facilitating long-range interchain tunneling.

## Conclusions and Outlook

We expect the implications of our work extend across a wide range of CP systems and applications. The ability to arbitrarily dilute CPs to any desired conductivity without extreme drop-offs in mobility provides utility in and of itself. For instance, organic neuromorphic devices require extremely low conductances (<10^–8^ S) while operating at high switching speeds (>1 MHz).^32^ By preserving the mobility as the CP is diluted, the required charge modulation (*i.e.*, capacitance) for switching can remain low, lowering the RC charging time^45^ relative to much lower mobility CPs.

Our results also elucidate why displacement of charge carriers in OECTs can occur rather efficiently. While electronic and ionic charges are often discussed as residing in the crystalline and amorphous region of the OMIEC, respectively, during electrochemical (de)doping, ions and holes must interact electrostatically. We expect that, for PEDOT:PSS, the excellent electronic transport properties of the ionically conductive phase allow for electronic and ionic charges to couple rapidly, which is crucial in electrochemical gating of OMIECs. For example, Rivnay *et al.* demonstrated that reduction of PEDOT chains during electrochemical gating occurs much faster for chains within the ionically conductive PSS-rich region compared to aggregated PEDOT-rich regions.^11^ This rapid reduction requires efficient electronic charge transport out of the ionically conducting phase.

Finally, our demonstration of p(g2T-TT) blended with aromatic and non-aromatic polymers reveals that the findings of this work are applicable to CPs beyond PEDOT:PSS. We expect further design and development of CPs, OMIECs, and blends to benefit from optimization of electronic tunneling through non-conductive (or in the case of OMIECs, ionically conductive) phases by improving the tunneling efficiency through the side chain/ionically conductive polymer through the inclusion of aromatic functional groups.

From a fundamental standpoint, the results in this work expand the general mechanistic understanding of charge transport in CPs. The results reveal that efficient tunneling through PSS enables the high electronic conductivities and mobilities observed for PEDOT:PSS films. Furthermore, the combination of our experimental methods and multiscale tunneling model provides a framework for assessing the transport properties of the solubilizing component in other CP systems. We expect this framework to be broadly applicable to CPs, which rarely consist of purely conducting or insulating phases, but instead consist of mixed phases containing both conducting and insulating constituents. While the relationship between CP morphology and charge transport properties have long been the subject of intense study, we assert that the role of solubilizing components on interchain transport, which often acts as a transport bottleneck,^9^ is overlooked and merits closer investigation.

## Supporting Information Available

Experimental methods, narrowing down the possible transport mechanisms, Monte Carlo simulations, MD simulations, CT network generation, transport simulations, and average tunneling distance calculation (PDF)
