# Supplementary material for: Efficient Electronic Tunneling Governs Transport in Conducting Polymer-Insulator Blends
Source: J Am Chem Soc. 2022 Jun 6;144(23):10368–76. doi: 10.1021/jacs.2c02139 (PMC9204759; doi:10.1021/jacs.2c02139)
Supplement: Supplementary file 1 — ja2c02139_si_001.pdf [file ja2c02139_si_001.pdf]

Supporting Information

Efficient electronic tunneling governs transport in conductive polymer-insulator blends

Scott T. Keene,[*1,†] Wesley Michaels,[2] Armantas Melianas,[1,‡] Tyler J. Quill,[1] Elliot J. Fuller,[3] Alexander Giovannitti,[1] Iain McCulloch,[4] A. Alec Talin,[3] Christopher J. Tassone,[5] Jian Qin,[2] Alessandro Troisi,[*6] Alberto Salleo[*1]

[1] Stanford University Department of Materials Science and Engineering, Stanford, CA 94305, United States
[2] Stanford University Department of Chemical Engineering, Stanford, CA 94305, USA
[3] Sandia National Laboratories, Livermore, California 94551, United States
[4] University of Oxford Department of Chemistry, Oxford OX1 3TA, United Kingdom
[5] Stanford Synchrotron Radiation Light Source, SLAC National Accelerator Laboratory, Menlo Park, CA 94025, United States
[6] University of Liverpool Department of Chemistry, Liverpool L69 3BX, United Kingdom

Experimental methods

Preparation of mixed conducting polymer blends
PEDOT:PSS (Hereaus, Clevios PH 1000, 1.3 wt.%) aqueous solution was prepared by adding 6 vol.% ethylene glycol (Sigma Aldrich) as a morphology enhancer and 0.1 vol.% dodecylbenzene sulfonic acid (Sigma-Aldrich) as an adhesion promoter. A separate solution of PSS:Na was prepared by dissolving 70,000 Da molecular weight PSS:Na (Sigma Aldrich) into deionized water to obtain a 1.3 wt.% solution. The PEDOT:PSS and PSS:Na solutions were combined to achieve varied dilution ratios and sonicated for 10 minutes to achieve a homogeneous dispersion. 1 vol.% (3-glycidyloxypropyl)trimethoxysilane (Sigma-Aldrich) was added to the diluted PEDOT:PSS mixtures as a crosslinking agent to reduce water solubility. Solutions were filtered through a 0.45 µm PVDF filter (Fisher Scientific) prior to spin coating. Poly(2-(3,3′-bis(2-(2-(2-methoxyethoxy)ethoxy)ethoxy)-[2,2’-bithiophen]-5-yl)thieno[3,2-b]thiophene) (p(g2T-TT)), polyethylene glycol (PEG, M_n = 400 Da), and poly(4-methoxystyrene) (p(4MeOS), M_n = 800 Da) were each dissolved in chloroform at a concentration of 3 mg mL^{-1} and stirred overnight. For diluted p(g2T-TT):PEG, p(g2T-TT) solutions were mixed with PEG solutions in 1:1 and 1:2 volume ratios, and for p(g2T-TT):p(4MeOS), p(g2T-TT) solutions were mixed with p(4MeOS) solutions in 1:1, 1:2, and 1:4 volume ratios.

Device Fabrication
Van der Pauw device photolithographic patterning was performed on silicon wafers with a 200 nm thermal oxide. First, metal interconnects (5/100 nm Ti/Au) were patterned using a standard lift-off process. The wafers were subsequently coated with 1.5 µm of Parylene-C as the insulating layer, which was crosslinked using a treatment of 3-(trimethoxysilyl)propyl methacrylate to promote adhesion to the substrate. Following the first Parylene-C layer, a dilute soap (3% Micro-90 in deionized water) solution was spin-cast on top, followed by deposition of a second Parylene-C layer, later used as a peel-off layer. The wafers were then coated with 75 nm of Ti using e-beam evaporation, photolithographically patterned and dry etched to define areas for the mixed conducting polymer. The wafer dies were cleaned with isopropanol sonication followed by ultraviolet–ozone cleaning before spin-coating the polymer layer. Diluted PEDOT:PSS solutions were spun on the wafer die at 1000 RPM for 2 min and subsequently baked at 120 °C for 20 min. The top Parylene-C layer was then peeled off to retain the polymer film only in the photolithographically defined areas. The wafer dies were gently rinsed in deionized water to eliminate residual soap and were subsequently dried at 120 °C for 5 min.

Organic electrochemical transistor fabrication consisted of evaporating 5 nm Cr/50 nm Au on clean glass substrates (1 inch by 1 inch) using a stainless-steel shadow mask to define the channel area. Next, the mixed conducting polymer blends were spin-coated on gold-coated glass slides at 1000 RPM for 2 min and baked 20

min (120 °C for PEDOT:PSS blends, 60 °C for p(g2T-TT) blends). Polymer was removed from the substrate with methanol to define 1 mm by 1 mm channels. For PEDOT:PSS blends, a polydimethylsiloxane (PDMS) well (2 mm diameter) was fixed to the substrate to contain the aqueous electrolyte solution (100 mm NaCl). For p(g2T-TT) samples, a 5 mm by 5 mm gate was also defined and a solid electrolyte made up of 80 wt.% EMIM:TFSI and 20 wt.% PVDF-HFP was dissolved in acetone (45 mg mL^{-1}) and deposited onto the channel/gate using drop casting. For cyclic voltammetry and UV–vis–NIR measurements, samples were coated onto transparent indium-tin oxide (ITO) coated glass slides using the same conditions as above. For EIS, samples were spin-coated on clean ITO coated slides and the electrode area was defined with a circular PDMS well defined with a biopsy punch (r = 2 mm). For GIWAXS, GISAXS, and c-AFM measurements, samples were spin-coated on n-doped silicon (2 cm by 2 cm) with a native oxide layer using spin speeds of 1000 RPM, 1000 RPM, and 2000 RPM, respectively.

Transistor Characterization

Transfer curves of PEDOT:PSS blends were measured with a Keithley 2612B source-measure unit with custom LabView code using a silver/silver chloride (Ag/AgCl) pellet as the gate electrode. The drain voltage V_D was set to −0.6 V and the gate voltage V_G was swept from 0.7 or 0.6 V to −0.6 V and back with voltage steps of 0.01 V. Transfer curves of p(g2T-TT) blends were measured with the same instrumentation with a large (5 mm by 5 mm) p(g2T-TT) film as the gate electrode. The drain voltage V_D was set to −0.6 V and the gate voltage V_G was swept from 0.7 V to −0.7 V and back with voltage steps of 0.01 V. Material mobilities for diluted PEDOT:PSS and p(g2T-TT) were measured using a previously reported current pulsing technique[1] where a constant gate current I_G is applied and the drain current I_D is monitored (**Figure S3**). The linear slope of the drain current vs. time dI_D/dt is proportional to I_G/t_h, where t_h is the time-of-flight for holes across the length L of the channel ($\mu = L^2\ t^{-1}\ V_D^{-1}$).

Temperature dependent conductance measurements

Temperature dependent conductivity measurements were performed using a vacuum probe station (MMR technologies) and two source-measure units (Keysight) with custom LabView code. The sample was cooled using Joule-Thompson cooling with high pressure nitrogen (99.999% purity, Praxair). A temperature controller (MMR K-20) was used to regulate the temperature of the sample and Van der Pauw resistivity measurements were carried out as described in **Figure S5**.

GIWAXS and GISAXS Measurements

Grazing incidence wide-angle x-ray scattering (GIWAXS) experiments were carried out at Stanford Synchrotron Radiation Lightsource (SSRL) at beamline 11-3 using an area CCD detector (Rayonix MAR 225) at a distance of 316.386 mm (calibrated with LaB6 standard reference), an incident beam energy of 12.73 keV, and an incidence angle of 0.1°. GI-small-angle x-ray scattering (GISAXS) experiments were carried out at SSRL at beamline 1-5 using an area detector (Rayonix 165 CCD Camera) at a distance of 2879.14 mm (calibrated with AgBe standard reference), an incident beam energy of 12 keV, and an incident angle of 0.14°. For all x-ray scattering measurements, the beam path was filled with helium to avoid air scattering between the sample and detector. The data reduction from 2D to 1D data and fitting was performed using Nika [2] and WAXStools.[3]

Thickness Measurements

Sample thicknesses were measured using a Bruker Dektak XT profilometer with 1 mg of force and a scan rate of 35 µm s^{-1}. Sample thickness was averaged over five individual measurements at different locations of the film.

Conductive atomic force microscopy

Conductive atomic force microscopy was carried out using PeakForce tapping mode in a Bruker Icon microscope. The probes were coated with a Pt-Ir alloy and had a 25 nm radius of curvature. Voltages of 2 V were applied between the tip and sample and the current passing through the tip was measured with a transimpedance amplifier.

Transport simulations

PEDOT:PSS films are composed of PEDOT-rich regions immersed in a PSS-rich matrix.[4] The multi-scale model for charge transport developed in this work follows this characterization at three length scales: the molecular scale (**Figure 4b**) models transport between individual PEDOT molecules, the mesoscale (**Figure 4c**) models transport between PEDOT-rich regions, and the macroscale (**Figure 4d**) models transport across the entire film.

At the molecular scale, we assume that PEDOT-rich regions themselves are highly conductive compared to the PSS-rich matrix due to their higher concentration of PEDOT. In contrast, hole transport through the PSS matrix is slow: we assume that charges tunnel between isolated clusters of PEDOT oligomers in this region, with PSS molecules acting as bridging sites for super exchange. The rate of this process is represented as:

$$k_{\mathrm{ET}}(R_{\mathrm{nn}}) = k_0 \exp(-\beta R_{\mathrm{nn}}) \qquad (S1)$$

where k_0 [sec^{-1}] is an adjustable prefactor, β [Å^{-1}] is the tunneling attenuation factor of PSS, and R_{nn} [Å] is the nearest-neighbour distance between the oligomers [5]. We test $0.3\ \text{Å}^{-1} \leq \beta \leq 1.0\ \text{Å}^{-1}$ in this work, which is representative of values found for bridging molecules with either alkyl or aromatic substituents.[5-6] Because the tunneling distance is similar to the length of PEDOT oligomers, we use atomistic molecular dynamics (MD) simulations to generate morphologies of the PSS-rich matrix. Rod-like and point-like geometries are also tested (**Figure 4f**, and **Section S5**).

At the mesoscale, we model a charge-transfer (CT) network composed of two PEDOT-rich grains and PEDOT clusters in the surrounding PSS matrix. A steady-state CT rate, $k_{\mathrm{ss}}(R;X)$, between the two grains is calculated (**Section S4**) as a function of R, the center-center grain separation distance, and X, the PEDOT to PSS weight ratio.

At the macroscale, we model a CT network where PEDOT-rich grains mediate transport across a PEDOT:PSS film. Film morphologies are generated via MC simulation (**Section S2**), in which PEDOT-rich grains are modelled as hard spheres immersed in a cubic box. We assume that CT rates between PEDOT-rich grains can be determined pairwise, *i.e.* that the presence of a third grain does not affect CT between the first two. The pairwise CT rate is interpolated from $k_{\mathrm{ss}}(R;X)$. Mobility is calculated (**Section S5)** as a function of PEDOT:PSS weight ratio by simultaneously varying the concentration of PEDOT in the PSS-rich phase and the concentration of PEDOT-rich grains in the film. **Table S1** contains the parameters used for each weight ratio.

Atomistic simulations

MD simulations are conducted in GROMACS,[7] using parameters developed by us for highly doped PEDOT[8-9] and Generalized AMBER Force Field parameters for PSS.[10] MD and MC simulations are further detailed in **Sections S2** and **S3**. The MDAnalysis[11] python package aided in analysis. Simulation images are generated with the Visual Molecular Dynamics (VMD) software.[12] Detailed procedural information for all modelling is found in **Sections S2-S6**.

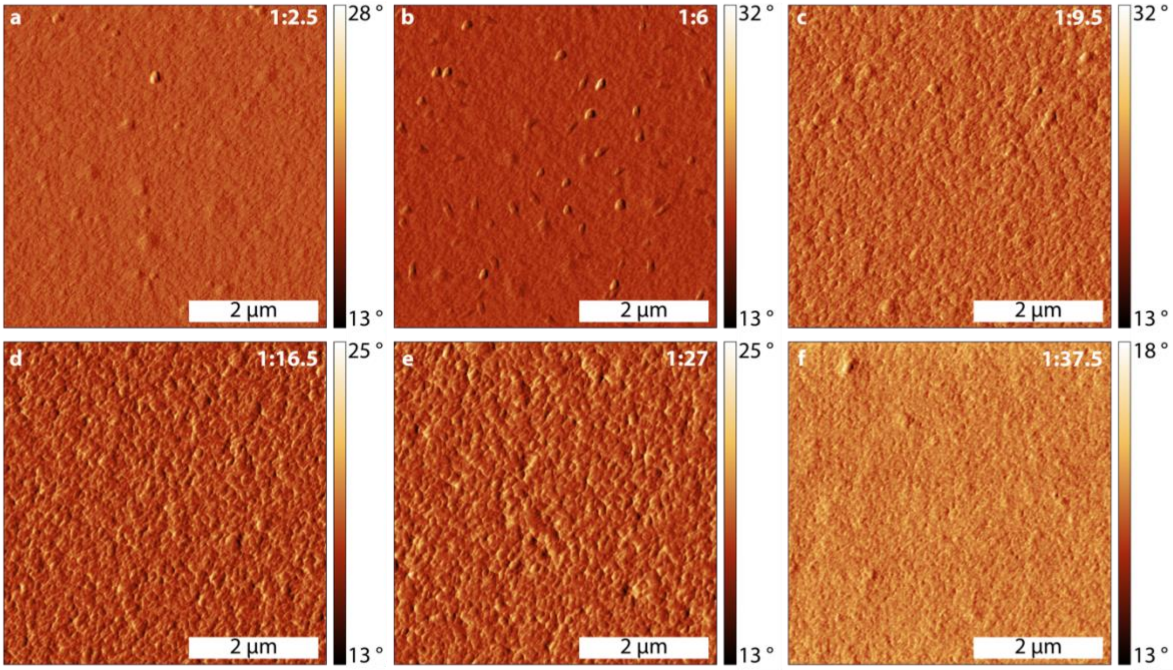

Figure S1: Phase contrast AFM images. (a-f) Phase contrast AFM images for diluted PEDOT:PSS thin films (dry) ranging from 1:2.5 to 1:37.5 weight ratio of PEDOT:PSS showing the uniformity of the thin film surface with some large particles on the order of the filter size (*ca.* 0.45 µm) present in the **(a)** 1:2.5 and **(b)** 1:6 weight ratio samples.

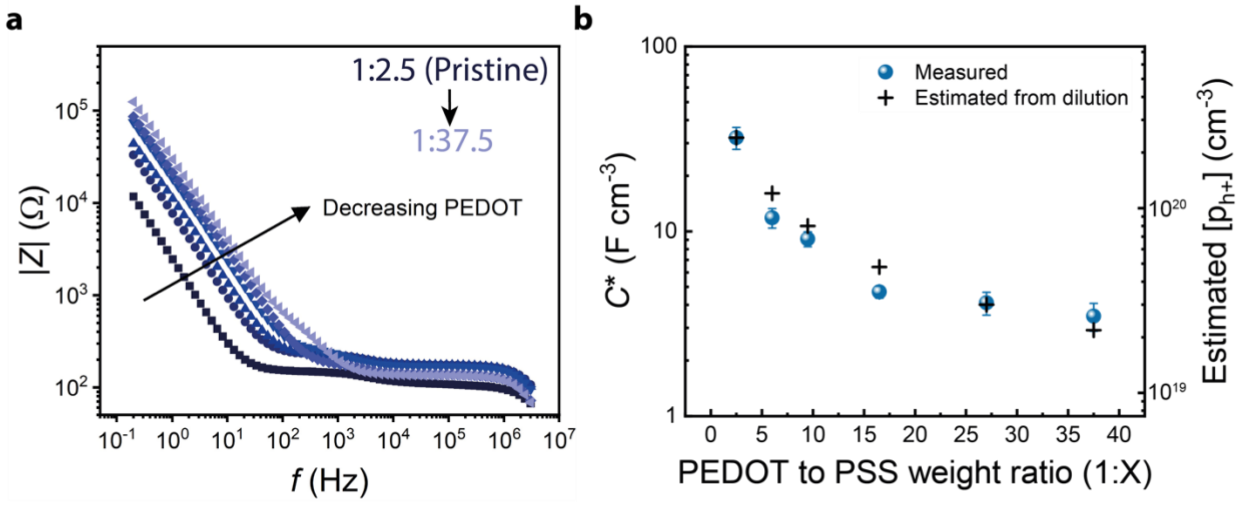

Figure S2: Volumetric capacitance and estimated carrier concentrations of diluted PEDOT:PSS blends. (a) Electrochemical impedance spectra taken with 100 mM sodium chloride aqueous electrolyte for PEDOT:PSS blends and **(b)** volumetric capacitances (blue circles) extracted from the low frequency regime of **(a)** compared to estimated capacitance (black plus signs), where the expected capacitance (C_{expected}) is calculated as:

$$C_{\text{expected}} = \frac{m_{\text{PEDOT,diluted}}}{m_{\text{PEDOT,pristine}}} C_{\text{pristine}}$$

where m is the mass fraction of PEDOT and C_{pristine} is the capacitance of the 1:2.5 weight ratio PEDOT:PSS.

Volumetric capacitances were used to estimate charge carrier densities of dry films and the values are shown on the left axis of **(b)**. For PEDOT:PSS, the volumetric capacitance arises from ionic charges displacing electronic charges,[13] and thus the capacitance is intimately tied to the charge carrier concentration. The volumetric capacitance of PEDOT:PSS is approximately constant with voltage once the potential exceeds the oxidation onset which is *ca.* -0.9 V *vs.* Ag/AgCl.[14] Therefore, we estimate the carrier density as the amount of charge which is stored in the PEDOT:PSS per unit volume when swept from the oxidation onset to the open-circuit potential (OCP, +0.3 V *vs.* Ag/AgCl) of as-cast PEDOT:PSS. We use the potential difference between the OCP and oxidation onset (1.2 V) multiplied by the constant C^* to estimate hole densities, $[p_{h+}]$, in the diluted PEDOT:PSS samples with the following equation:

$$[p_{h^+}] = 1.2\ V * C^* * \frac{1}{e}$$

where e is the elementary charge.

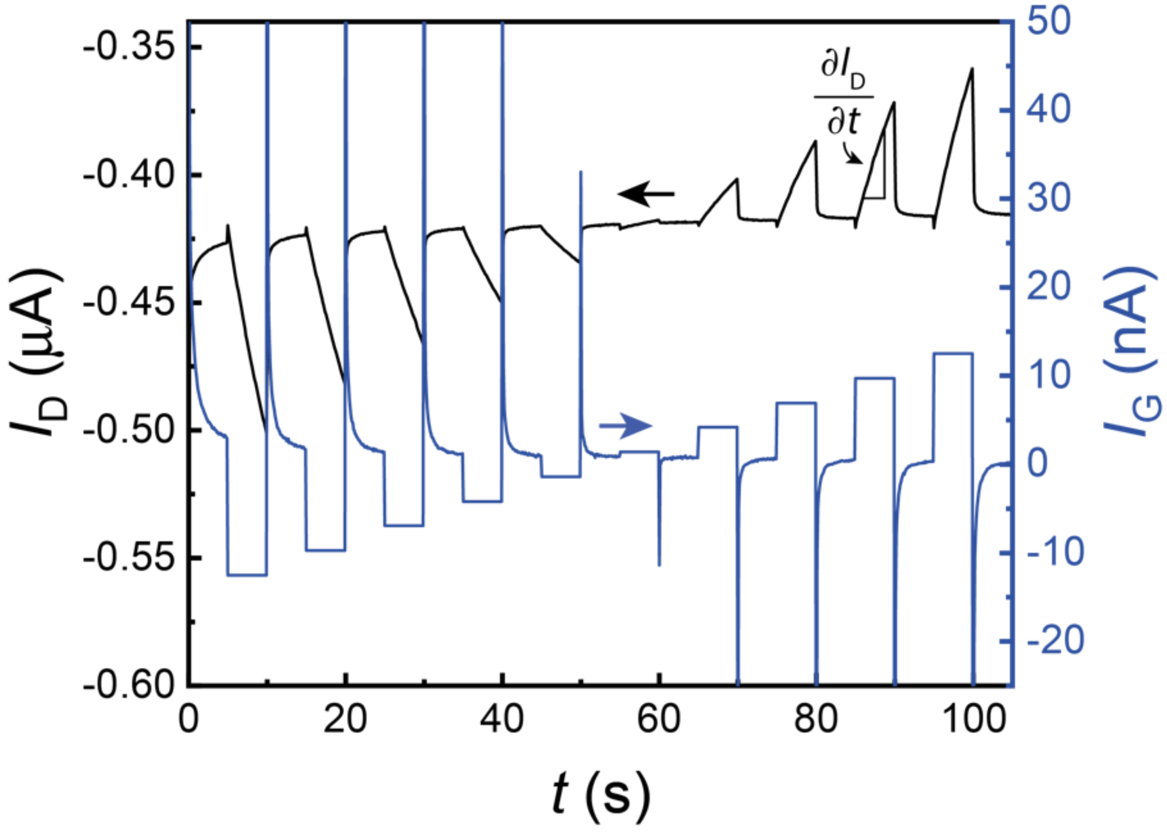

Figure S3: Mobility measurement technique. Hole mobility measurement using current pulsed technique. Hole mobilities are measured by applying a constant source-drain bias V_D of -0.1 V and monitoring the change in source-drain current I_D (black, left axis) in response to applied gate current I_G (blue, right axis) pulses. The hole mobility μ is given by:[1]

$$\mu = \frac{\partial I_D}{\partial t} * \frac{L^2}{V_D * I_G}$$

where $\partial I_D/\partial t$ is the slope of I_D *vs* time t and L is the length of the OECT channel in cm. Thus, μ can be extracted by fitting the slope of I_D *vs. t* during a single current pulse. To ensure an accurate measurement of μ, we used a pulse train of 10 different values of I_G (as shown above). The results from all 10 pulses (positive and negative) were averaged.

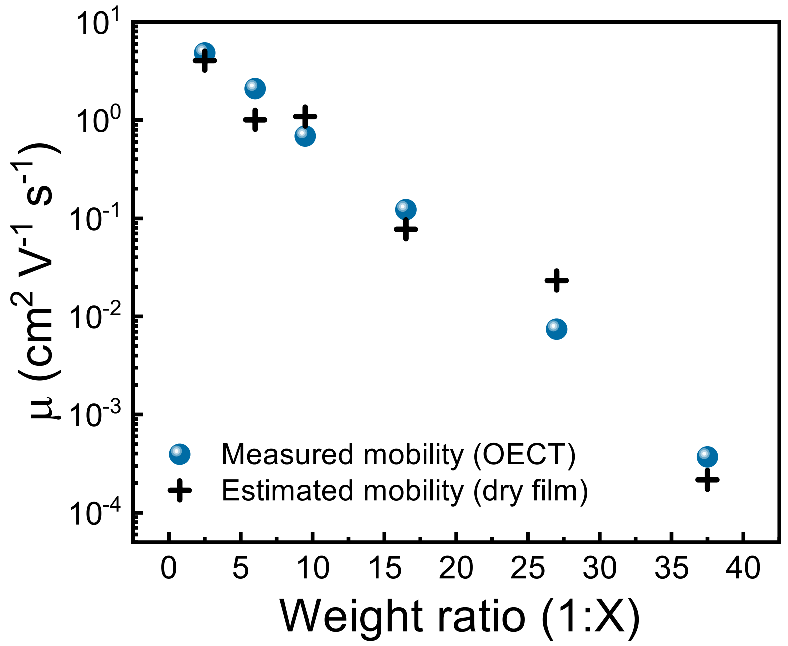

Figure S4: Comparison between measured and estimated mobilities in wet and dry films. Measured mobility values (blue circles) using organic electrochemical transistors (OECTs) as described in **Figure S3** compared to estimated mobility values of dry films under vacuum at room temperature (black plus signs). Hole mobilities, μ, for the dry films are estimated from the dry film conductance, σ_s using the estimated charge carrier concentration, $[p_{h^+}]$, calculated from the volumetric capacitance as described in **Figure S2**, yielding the following equation:

$$\sigma = e * \mu * [p_{h^+}]$$

where e is the elementary charge (1.602 × 10^{-19} C). Rearranging, we can solve for the hole mobility as follows:

$$\mu = \frac{\sigma}{e * [p_{h^+}]}$$

The agreement between the mobilities measured using OECTs and mobilities estimated for the dry films indicates that the transport in both dry and hydrated films follow a similar process.

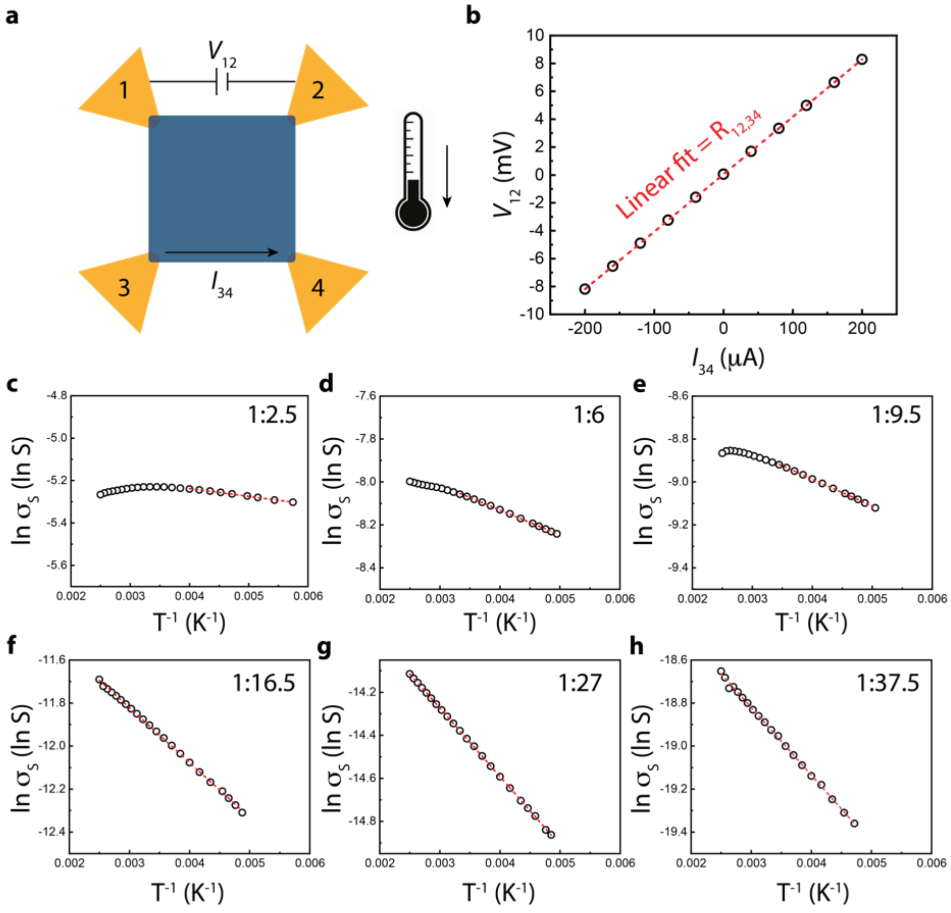

Figure S5: Temperature-dependent conductivity measurements. **(a)** Schematic of the Van der Pauw device structure showing the applied current, I_{34}, and the measured voltage, V_{12}. **(b)** Current is swept linearly (range varies depending on the sample) and the voltage is plotted. A linear fit of the curve gives the resistance, $R_{12,34}$, and for each temperature both $R_{12,34}$ and $R_{34,12}$ are measured and averaged to get R. The sheet conductance is calculated using:

$$\sigma_S = \frac{1}{R_S} = \frac{\ln(2)}{\pi R}$$

where R_S is the sheet resistance. Panels **(c-h)** show plots of the natural logarithm of sheet conductance as a function of temperature (black circles) for each sample with linear fits (red dotted lines) used to fit the transport activation energy, E_A, and pre-factor, $\sigma_{s,0}$. E_A is calculated from the slope of the Arrhenius plots using:

$$-E_A = \text{slope} * k_B$$

Where k_B is the Boltzmann constant (8.617×10^{-5} eV K^{-1}). Additionally, $\sigma_{s,0}$ is calculated from the y-intercept of the linear fit using the following equation:

$$\sigma_{s,0} = \exp(\text{y-intercept})$$

The hole mobility μ of the dry films can be estimated as described in **Figure S4** using the estimated charge carrier density $[p_{h+}]$. Similarly, μ_0 is calculated using the sheet conductance pre-factor $\sigma_{s,0}$ with the following equation:

$$\mu_0 = \frac{\sigma_{s,0}}{e * [p_{h^+}] * t}$$

Where t is the film thickness in cm and e is the elementary charge (1.602×10^{-19} C). The resulting μ_0 values are plotted in **Figure 2d** in the main text.

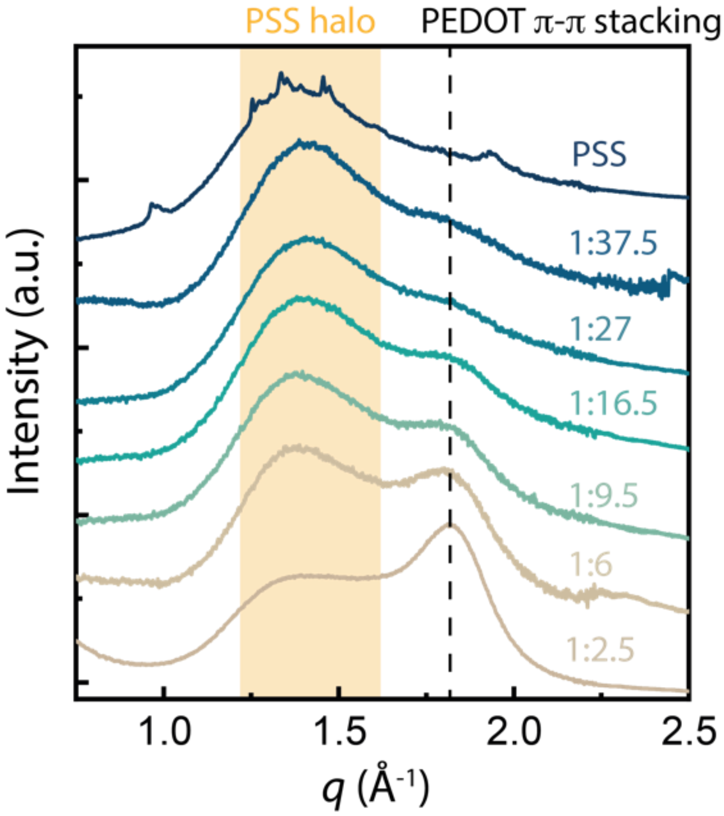

Figure S6: Grazing-incidence wide-angle x-ray scattering of PEDOT:PSS blends. Normalized x-ray scattering intensity for GIWAXS showing a decrease in the p-p stacking peak intensity of PEDOT relative to the amorphous halo from scattering of PSS. The presence of the p-p stacking shoulder even at low PEDOT concentrations shows that there are PEDOT-rich aggregates present in the film at high dilution ratios. Plots are the intensity of the GIWAXS pattern integrated from and azimuth angle of -10° to 10° and normalized the thickness of each film. The plots are shifted along the y-axis for comparison.

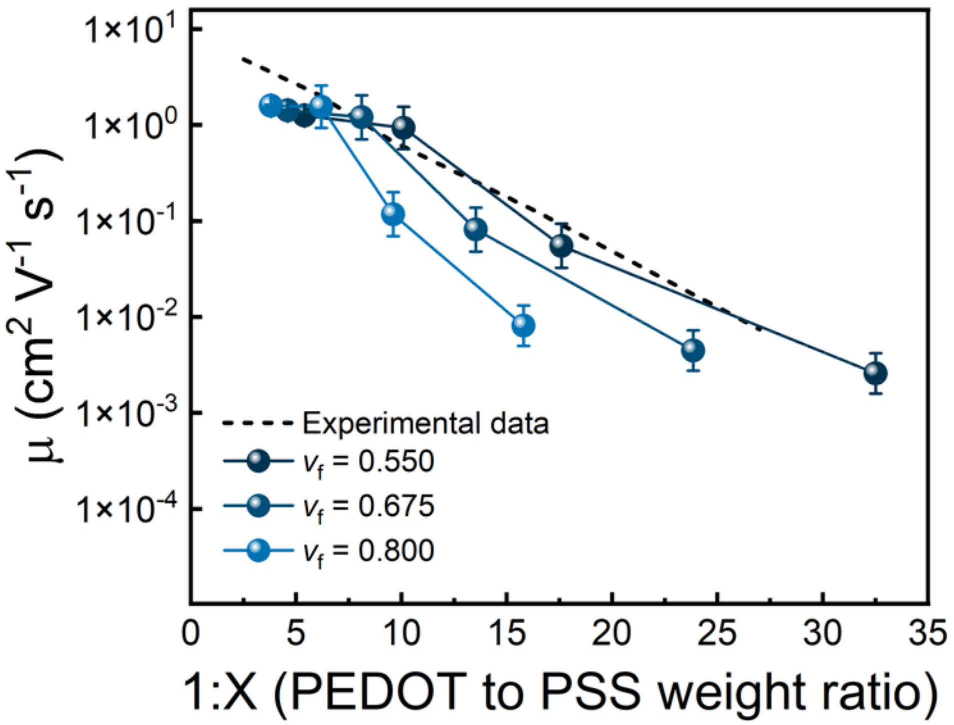

Figure S7: Effect of grain volume fraction in pristine PEDOT:PSS on mobility. Mobility *vs.* dilution relationships with different initial grain volume fractions, v_0. To assess the sensitivity of the model to this parameter, we test a subset of the range [0.55, 0.8] postulated by Rivnay *et al.*[4]

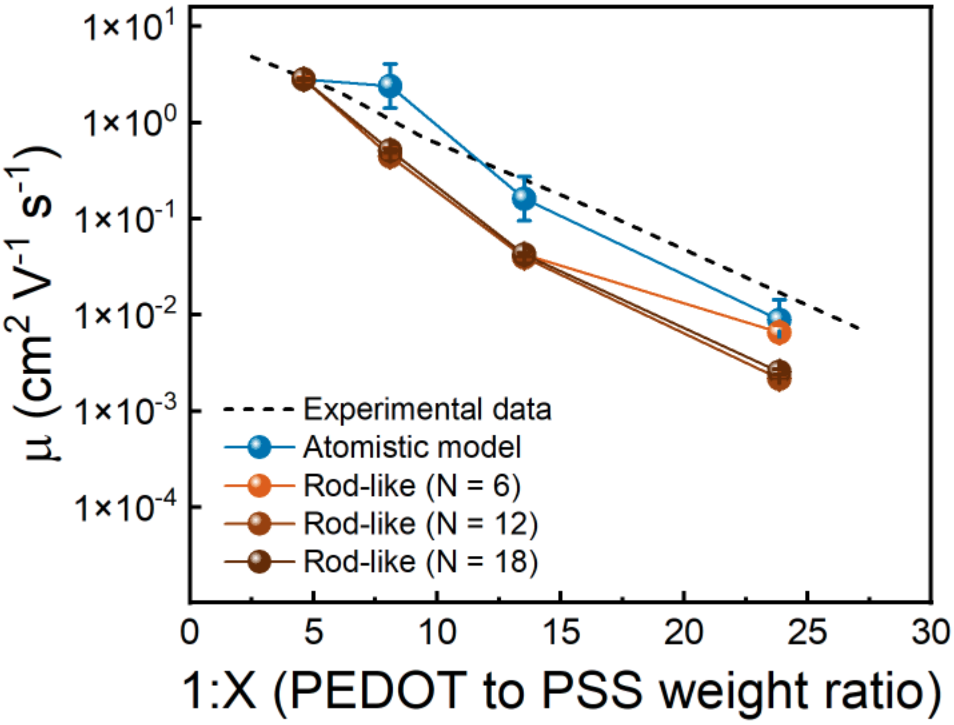

Figure S8: Effect of rod-like particle length on mobility. CT simulations were performed with rod-like particle of lengths 23.3 Å, 46.6 Å, and 69.9 Å, corresponding to a rigid rod approximation of PEDOT oligomers of length N = 6, 12, and 18, respectively. The equivalent PEDOT concentration was held equal to the concentration in atomistic simulations (blue trace) by adjusting the number of rods in each CT simulation. At high concentrations, long rods yield higher mobilities than do shorter rods because intramolecular transport is instantaneous in the model. At low concentrations, the relatively higher number of smaller rods reduces tunneling distances compared to longer rods, increasing hole mobility. The traces are shifted vertically using an arbitrary prefactor for comparison.

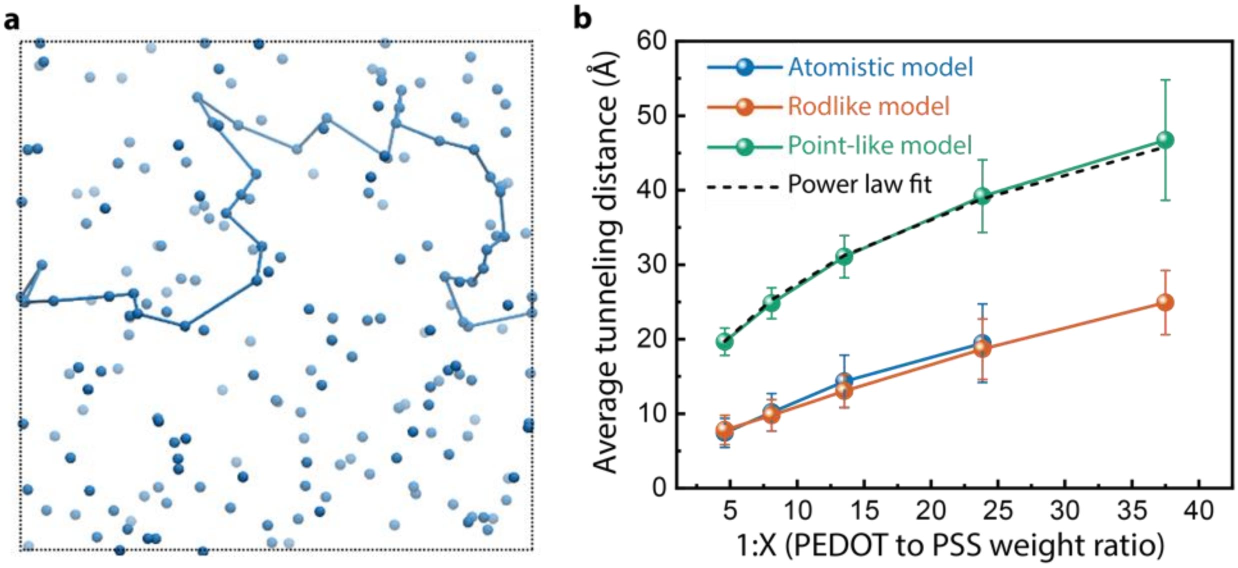

Figure S9: Average tunneling distance. (a) Fastest charge transfer path (blue line) through a configuration of point-like particles (blue spheres) at a PEDOT to PSS weight ratio of 1:37.5. The simulation box is cubic with side length 425 Å. **(b)** Average tunneling distance *vs.* PEDOT to PSS weight ratio for atomistic (blue), rodlike (red), and point-like (yellow) particle representations. Error bars denote a 95% confidence interval of the mean over (N = 15 for atomistic samples, N = 50 for rodlike and point-like samples). See **Section S6** for details.

Table S1: Model parameters for CT simulations with varying initial grain volume fractions.

Initial grain volume fraction, v_0	PEDOT:PSS weight ratio in film†	Volume fraction of PEDOT-rich grains‡	Number of PEDOT-rich grains§	PEDOT:PSS monomer ratio in PSS-rich matrix
0.55	1:5.4	0.18	809	1:8.7
	1:10.1	0.10	469	1:16.8
	1:17.6	0.06	280	1:29.9
	1:32.5	0.03	155	1:56.0
0.675*	1:4.6	0.25	1141	1:6.5
	1:8.1	0.16	702	1:13.7
	1:13.5	0.10	440	1:25.7
	1:23.9	0.06	257	1:49.2
0.8	1:3.8	0.35	1574	1:6.0
	1:6.2	0.23	1058	1:10.3
	1:9.6	0.16	717	1:16.7
	1:15.8	0.10	451	1:28.5

† PEDOT:PSS and pure PSS densities of 1.01 and 0.80 g/cm^3 are assumed, respectively. A cubic film of size (266.3 nm)^3 is considered.

‡ To obtain these figures, we assume that PEDOT-rich grains have a 55%, 67.5%, and 80% volume fraction, respectively, in a film with a PEDOT:PSS weight ratio of 1:1.5. We assume that the PEDOT concentration within the PEDOT-rich domains stays constant when the overall film is diluted with PSS, but that their concentration is diluted as PSS is added. **Figure S7** shows $\mu(X)$ results for initial volume fractions of 55%, 67.5%, and 80%.

* An initial grain volume fraction of 67.5% was used for the model in **Figure 4e** based on experimental results from ref. 4.

§ PEDOT-rich domains are assumed to be spheres of diameter 20 nm with equal molar concentrations of PEDOT and PSS.

Table S2: PEDOT:PSS monomer ratios sampled in atomistic simulation and corresponding conditions.

PEDOT:PSS monomer ratio in PSS-rich matrix	Number of PEDOT chains (N=6)	Number of PEDOT chains (N=12)	Number of PSS chains (N=25)	PEDOT weight fraction	Cubic simulation box length (nm)
1:6.5	21	21	96	0.095	8.56
1:13.6	10	10	96	0.048	8.43
1:25.5	5	5	96	0.026	8.36
1:49.2	3	3	96	0.014	8.34

Table S3: MD simulated annealing protocol

Annealing step	Description	Temperature	Simulation time
1	Fluidize sample above T_g	1100 K	10 ns
2	Cool to below T_g	1100 K -> 800 K (linear ramp)	2 ns
3	Hold below T_g	800 K	10 ns
4	Cool to room temperature	800 K -> 300 K (linear ramp)	2 ns
5	Hold at room temperature (for sampling)	300 K	1 ns
6 (optional)	Ramp back to high temperature to repeat sampling, if needed	300 K -> 1100 K (linear ramp)	2 ns

Section S1: Narrowing down the possible transport mechanisms

To develop a transport model consistent with the experimental data collected in this work, we first examined previously reported descriptions of transport in PEDOT:PSS and other conducting polymers (CPs). From structural results, we conclude a granular structure (which is consistent with previous studies)[4, 15] which consists of conductive grains dispersed in a less conductive matrix (**Figure 3a-c**). Thus, we explored mechanisms by which charges can transport between conducting grains and how that is affected by the dilution of PEDOT by addition of PSS.

First, we considered mechanisms which would allow direct transport (single hop) between individual conductive grains. Based on estimations of average particle spacing (**Section S2**) we expect the inter-grain distances to range from 1 nm to 4 nm for the pristine PEDOT:PSS (1:2.5) up to 25 nm to 60 nm for the most diluted samples. While direct tunneling between grains (which drops-off exponentially with increased distance) would approximately capture the exponential drop-off in mobility with increased grain spacing, this would result in unrealistic tunneling attenuation coefficient of β < 0.03 $Å^{-1}$ for the matrix. Other models predict that transport between conductive grains in the CP microstructure are dominated by the presence of tie chains[16] which bridge between grains. However, the typical molecular weight, M_W, of PEDOT chains in PEDOT:PSS dispersions is quite low, on the order of 1000 to 2500 g mol^{-1}, which corresponds to *ca.* 7 to 17 monomer units[17] or a length of *ca.* 3 nm to 7 nm. Thus, while tie chains may contribute to transport at the highest PEDOT concentrations, they are not expected to affect transport in the further diluted samples.

We also considered whether we could model the transport using a previously reported generalized effective media theory[18] (GEMT) which treats the conductivity of the mixture as a composite of the individual components (PEDOT or PEDOT:PSS at a particular concentration, and pure PSS) with some critical percolation threshold. However, the GEMT predicts the presence of a critical threshold for conduction, where for concentrations far above or below this threshold, the conductance is approximately constant with concentration. In contrast, we see that the conductance in our diluted PEDOT:PSS samples continues to drop with increasing dilution with a single scaling behavior over the entire range tested. Furthermore, we were unable to probe the electrical conductivity of pure PSS using the same measurement techniques due to the ultralow currents, indicating that PSS by itself is much more resistive than any of the blends examined.

Finally, we examined the use of percolation model to describe the transport scaling behavior of the diluted PEDOT:PSS films. In previous work, conduction in PEDOT:PSS has been described as a percolating system of nearly 1D conductive filaments dispersed in a nonconductive matrix.[19] While we conclude a granular structure of PEDOT:PSS based on our structural results (**Figure 3, Figure S1**), the presence of conductive filaments in the bulk of the PEDOT:PSS films cannot be entirely ruled out with the presented structural data. Instead, we rule out percolation based on the lack of a concentration linked to the critical percolation threshold for conduction. Nanocomposite materials containing conductive particles in insulating polymer matrices have been studied very extensively within the framework of percolation models,[20-21] and in all cases the electrical conductivity changes by several orders of magnitude changes for modest (few percent) changes of the conductive phase concentration. Furthermore, the conductivity saturates at concentrations above and below the percolation threshold. The diluted PEDOT:PSS samples display a continuous conductivity decay over a twenty-fold change in concentration, showing no indication of a critical concentration for conduction. In the absence of such critical points, the language of GEMT or percolation theory are not particularly useful and thus the frameworks are not used in the description of transport.

The weak temperature dependence of the transport process and exponential scaling indicates that the transport is limited by tunneling from one site to the next, where the population of available sites shows relatively low energetic disorder for occupation. The drop-off in tunneling rates is thus most likely due to spatial separation of sites, which are presumed to be PDEOT chains dispersed in the matrix surrounding conductive grains. Thus, in combination with the structural results (**Figure 3f**), we explored the scaling of charge transport for an increasingly diluted system where both the concentration (and therefore spatial separation) of conductive grains as well as the population of isolated PEDOT chains in the surrounding matrix are diluted (**Figure 4a-d**). This model presented the most reasonable match to the experimental results (**Figure 4e**).

Section S2: Monte Carlo simulations

We use Monte Carlo (MC) simulations to sample the positions of spherical PEDOT-rich grains in the film. Simulations begin by randomly initializing the positions of spheres in the box. Particles interact with a pairwise energy

$$E_{\mathrm{p}}(r) = 10\ \exp\left(-\frac{r - 2r_{\mathrm{s}} - 20\text{Å}}{35\text{Å}}\right) k_{\mathrm{B}}T \qquad (S2)$$

where r is the distance between the centers of the spheres and r_s is the radius of each sphere (100 Å).[15] The potential is repulsive at short ranges and becomes smaller than the thermal energy, $k_{\mathrm{B}}T$, when $r > 3r_s$. Thus, for the size of spheres used, this potential resembles a hard-sphere potential. The total energy in the simulation is:

$$E = \sum_{j=1}^{N} \sum_{i<j} E_{\mathrm{p}}(\boldsymbol{R}_j - \boldsymbol{R}_i) \qquad (S3)$$

where N is the number of spheres and $\boldsymbol{R}_i$ and $\boldsymbol{R}_j$ are the positions of spheres i and j, respectively. Trial configurations, generated by random translations of single spheres, are either accepted or rejected using the Metropolis-Hastings criterion. Each MC simulation consisted of 10^6 trial moves and we performed 24 simulations for each PEDOT:PSS weight ratio to improve the reproducibility of the results.

Section S3: Molecular Dynamics simulations

Based on the literature, we expect the oligomer length of PEDOT chains to range from N = 7 units to N = 17 units.[17] For computational simplicity, we approximated the distribution of PEDOT with equal concentrations of oligomers with N = 6 and N = 12 repeat units. Chains of length N = 18 were avoided to eliminate the possibility of self-interactions. PSS oligomers were of length N = 25. This value is many times the experimentally determined persistence length of 0.9 nm (4-5 monomers),[22] allowing for adequate configurational sampling. **Table S2** shows the number of PEDOT chains and PSS chains in MD simulations for different PEDOT concentrations in the PSS-rich phase. **Table S1** demonstrates how the number of PEDOT-rich regions used in transport simulations, combined with the PEDOT concentration in the PSS-rich regions, generates the desired experimental PEDOT:PSS weight ratios.

MD simulations are conducted in GROMACS[7] using parameters developed by us for highly doped PEDOT[8-9] and Generalized AMBER Force Field parameters for PSS.[10] Samples were initialized in the gas phase (density 0.035 g cm^{-3}) and compressed to a density of 1.36 g cm^{-3} with an algorithm based on that developed by Kong and Liu.[23]

Because PEDOT:PSS displayed a high glass transition temperature (T_g) in simulations (roughly 1050 K), sampling independent, equilibrated configurations at experimental temperatures is not feasible by running extended simulations at 300 K. Though running simulations at temperatures above T_g enhances sampling, rapid cooling of high-temperature simulations may kinetically trap films in unfavorable configurations. Zhang *et al.* showed that rapid quenching of fluidized $Cu_{64.5}Zr_{35.5}$ metallic glasses generates samples with low crystallinity and high internal energy.[24-25] To alleviate this concern, the authors designed a "sub-T_g annealing" protocol, wherein a fluidized sample is cooled to and held at a temperature near, but below T_g before being cooled to the final, desired temperature.[24] The protocol produced simulations with lower internal energy[24] and significantly greater amounts of experimentally observed clustering.[25] Thus, we adopted and optimized a similar sub-T_g annealing approach (**Table S3**) in our simulations.

GROMACS simulation files, including .mdp files for compression and annealing simulations, are hosted on GitHub. We repeated the algorithm in **Table S3** to gather five independent samples for each PEDOT:PSS weight ratio. The compression and annealing simulations for all weight ratios combined to a total of 750 ns of simulation.

Section S4: Charge transfer network generation

Charge transport at the mesoscale and macroscale is calculated as a steady-state rate through CT networks. Each network has a source, a drain, and intermediate nodes. At the mesoscale, one PEDOT-rich grain is the source, another is the drain, and clusters of PEDOT chains are intermediate nodes (**Figure 4c**). At the macroscale, the left edge of the film is the source, the right edge the drain, and PEDOT-rich grains are intermediate nodes (**Figure 4d**).

At the mesoscale, geometries were generated by concatenating periodic images of a single annealed MD snapshot (cubic unit cells approximately 8.5 nm in length). We used 3-7 images in the x-direction and 5 images in the y- and z-directions. We then placed two spherical PEDOT-rich regions on opposing faces of the box (along the x-axis). PEDOT molecules are grouped into a cluster if the nearest-neighbor distance between the thiophene backbones is within 5 Å. Pairs of backbones closer than 5 Å are assumed to be complexed without any interstitial PSS moieties, such that tunneling is not required for intermolecular CT within a cluster. Inter-cluster distances are calculated from the nearest-neighbor distance among all pairs of molecules in each cluster. **Equation S1** is used to calculate the CT rate from each pairwise distance element, forming the matrix of CT rates, $\boldsymbol{A}$, in **equation S4 (Section S5)**. Five MD samples were generated at each PEDOT to PSS weight ratio. Three pseudo-independent snapshots were generated by rotating each sample, yielding 15 total samples per PEDOT to PSS weight ratio.

For rod-like and point-like representations of PEDOT molecules, it is assumed that no clustering occurs. Therefore, the inter-cluster distance is equivalent to the inter-particle distance. Pairwise distances for rod-like molecules were determined using an algorithm by David Eberly.[26] We generated 50, 50, 250, and 250 samples with each representation for PEDOT to PSS weight ratios of 1:4.6, 1:8.1, 1:13.5, and 1:23.9 (**Table S2**), respectively.

At the macroscale, PEDOT-rich grain locations are generated via Monte Carlo simulation (**Section S2**). Center-to-center distances are calculated. Pairwise CT rates are interpolated directly from the $k_{ss}(R; X)$ relationship derived at the mesoscale. We sample 25 independent geometries for each PEDOT to PSS weight ratio. The configurational variety of PEDOT molecules at the mesoscale and PEDOT-rich grains at the macroscale give rise to the error bars in simulated mobility data.

Section S5: Transport simulations

All transport calculations were performed in MATLAB and are hosted on GitHub. For a network comprised of N nodes, a source, and a drain, steady-state CT transport can be described by the system of equations:[27]

$$\frac{d\boldsymbol{C}_{\mathrm{ss}}}{dt} = \boldsymbol{A}\boldsymbol{C}_{\mathrm{ss}} + \boldsymbol{J} = 0 \qquad (S4)$$

where $\mathbf{A}$ is an $(N+2) \times (N+2)$ matrix of CT rates, $\boldsymbol{C}_{ss}$ is a vector of the steady-state hole concentrations at each site, and $\boldsymbol{J}$ is the vector of the incident charge fluxes into each node. We assume that due to the presence of an electric field in the device, charge is injected into the matrix exclusively at the source and leaves exclusively at the drain. Thus, the $N+2$ elements of $\boldsymbol{J}$ are given by:[27]

$$J_k = J_0 \delta_{k0} \qquad (S5)$$

where J_0 is a proportionality constant with units of flux. The off-diagonal elements of $\boldsymbol{A}$ are determined using **equation S1** with the nearest-neighbor distances from simulation. Because no charge accumulates at steady state, the diagonal elements are $A_{ii} = \sum_{j=0}^{N+1}\left(1-\delta_{ij}\right)A_{ij}$. To ensure the drain is quickly draining, we set $A_{N+1,N+1} = \sum_{j=0}^{N} A_{N+1,j} - \max(\boldsymbol{A})$. Solving **equation S4** yields the steady-state CT rate,[27]

$$k_{ss} = \frac{J_0}{C_{ss_0}} \qquad (S6)$$

The specific value of J_0 does not affect the results because C_{ss_0} is proportional to J_0.

Section S6: Average tunneling distance calculation

In this section, we estimate the distances charges tunnel during device operation. Because charges may tunnel between any two PEDOT molecules, there are a vast number of CT pathways through the PSS-rich matrix. We inspect only the single fastest pathway to estimate the average tunneling distance, approximating that (1) CT through the fastest pathways generates most of the current in devices and (2) the fastest pathways will have similar intermolecular tunneling distances, r_{t}, due to the rapid decline in rate with increasing r_{t} (**equation S1**).

In this model, we calculate the average tunneling distance of the fastest path in an ensemble of PSS-rich matrix configurations:

$$\langle r_{\mathrm{t}}(X)\rangle = \langle \frac{\sum_{i=1}^{n} r_{t,i}^{*}}{n} \rangle \quad (S7)$$

where X is the film dilution, n is the number of steps in the fastest pathway in a configuration, and $r_{t,i}^{*}$ is the tunneling distance of the ith step of the fastest pathway. Here, $\langle \cdots \rangle$ represents an average over many independent configurations of the PSS-rich matrix. Configurations are (42.5 nm)^3 cubic cells generated as in **Section S4**, except that the PEDOT-rich grain size is set to zero to minimize boundary effects.

We calculate the CT time between pairs PEDOT molecules as

$$t_{\mathrm{ET}}(R_{\mathrm{nn}}) = \exp(\beta R_{\mathrm{nn}}) \quad (S8)$$

This is the inverse of **equation S1**, the CT rate. All pairwise CT times for a single configuration are combined into an array, where self-times are set to zero. The fastest pathway through a configuration is then identified with Dijkstra's algorithm[28-29] and the corresponding tunneling distances are used for statistical averaging in **equation S8**.

Figure S9 shows that $\langle r_{\mathrm{t}}(X)\rangle$ increases with dilution in the PSS-rich matrix. For atomistic and rodlike particles, $\langle r_{\mathrm{t}}(1{:}\,4.6)\rangle$ is comparable to our presupposed minimum tunneling distance of 5 Å (**Section S4**). At the maximal dilution, $\langle r_{\mathrm{t}}(1{:}\,37.5)\rangle$ ranges from 2.1 to 2.9 nm for rodlike particles. Given the close agreement between rodlike and atomistic particles from X = 1:4.6 to X = 1:23.9, we estimate the atomistic $\langle r_{\mathrm{t}}(1{:}\,37.5)\rangle$ using rodlike particles.

A power law scaling of $\langle r_{\mathrm{t}}\rangle \sim c^{1/3}$ is anticipated for pointlike particles, where c is the concentration of PEDOT molecules. Imagining PEDOT molecules arranged in a perfect cubic lattice with lattice constant a, the fastest paths will be along the edges of the lattice: $\langle r_{\mathrm{t}}\rangle = a$. Because $a \sim c^{1/3}$ from the geometry of the lattice, $\langle r_{\mathrm{t}}\rangle \sim c^{1/3}$. Indeed, the simulated data for point like particles (**Figure S9**, green trace) closely adhere to this trend (black dashed trace, with prefactor A fitted by regression). Rodlike and atomistic particles, having finite size, give shorter $\langle r_{\mathrm{t}}\rangle$ at all dilutions compared to pointlike particles.

References

1. Bernards, D. A.; Malliaras, G. G., Steady-State and Transient Behavior of Organic Electrochemical Transistors. *Advanced Functional Materials* **2007,** *17* (17), 3538-3544.
2. Ilavsky, J., Nika: software for two-dimensional data reduction. *Journal of Applied Crystallography* **2012,** *45* (2), 324-328.
3. Oosterhout, S. D.; Savikhin, V.; Zhang, J.; Zhang, Y.; Burgers, M. A.; Marder, S. R.; Bazan, G. C.; Toney, M. F., Mixing Behavior in Small Molecule:Fullerene Organic Photovoltaics. *Chemistry of Materials* **2017,** *29* (7), 3062-3069.
4. Rivnay, J.; Inal, S.; Collins, B. A.; Sessolo, M.; Stavrinidou, E.; Strakosas, X.; Tassone, C.; Delongchamp, D. M.; Malliaras, G. G., Structural control of mixed ionic and electronic transport in conducting polymers. *Nature Communications* **2016,** *7* (1), 11287.
5. Sayed, S. Y.; Fereiro, J. A.; Yan, H.; Mccreery, R. L.; Bergren, A. J., Charge transport in molecular electronic junctions: Compression of the molecular tunnel barrier in the strong coupling regime. *Proceedings of the National Academy of Sciences* **2012,** *109* (29), 11498-11503.
6. Huang, M.-J.; Hsu, L.-Y.; Fu, M.-D.; Chuang, S.-T.; Tien, F.-W.; Chen, C.-H., Conductance of Tailored Molecular Segments: A Rudimentary Assessment by Landauer Formulation. *Journal of the American Chemical Society* **2014,** *136* (5), 1832-1841.
7. Abraham, M. J.; Murtola, T.; Schulz, R.; Páll, S.; Smith, J. C.; Hess, B.; Lindahl, E., GROMACS: High performance molecular simulations through multi-level parallelism from laptops to supercomputers. *SoftwareX* **2015,** *1-2*, 19-25.
8. Michaels, W.; Zhao, Y.; Qin, J., Atomistic Modeling of PEDOT:PSS Complexes I: DFT Benchmarking. *Macromolecules* **2021,** *54* (8), 3634-3646.
9. Michaels, W.; Zhao, Y.; Qin, J., Atomistic Modeling of PEDOT:PSS Complexes II: Force Field Parameterization. *Macromolecules* **2021,** *54* (12), 5354-5365.
10. Wang, J.; Wolf, R. M.; Caldwell, J. W.; Kollman, P. A.; Case, D. A., Development and testing of a general amber force field. *Journal of Computational Chemistry* **2004,** *25* (9), 1157-1174.
11. Michaud-Agrawal, N.; Denning, E. J.; Woolf, T. B.; Beckstein, O., MDAnalysis: A toolkit for the analysis of molecular dynamics simulations. *Journal of Computational Chemistry* **2011,** *32* (10), 2319-2327.
12. Humphrey, W.; Dalke, A.; Schulten, K., VMD: Visual molecular dynamics. *Journal of Molecular Graphics* **1996,** *14* (1), 33-38.
13. Volkov, A. V.; Wijeratne, K.; Mitraka, E.; Ail, U.; Zhao, D.; Tybrandt, K.; Andreasen, J. W.; Berggren, M.; Crispin, X.; Zozoulenko, I. V., Understanding the Capacitance of PEDOT:PSS. *Advanced Functional Materials* **2017,** *27* (28), 1700329.
14. Keene, S. T.; van der Pol, T. P. A.; Zakhidov, D.; Weijtens, C. H. L.; Janssen, R. A. J.; Salleo, A.; van de Burgt, Y., Enhancement-Mode PEDOT:PSS Organic Electrochemical Transistors Using Molecular De-Doping. *Advanced Materials* **2020,** *32* (19), 2000270.
15. Nardes, A. M.; Kemerink, M.; Janssen, R. A. J.; Bastiaansen, J. A. M.; Kiggen, N. M. M.; Langeveld, B. M. W.; Van Breemen, A. J. J. M.; De Kok, M. M., Microscopic Understanding of the Anisotropic Conductivity of PEDOT:PSS Thin Films. *Advanced Materials* **2007,** *19* (9), 1196-1200.
16. Noriega, R.; Rivnay, J.; Vandewal, K.; Koch, F. P. V.; Stingelin, N.; Smith, P.; Toney, M. F.; Salleo, A., A general relationship between disorder, aggregation and charge transport in conjugated polymers. *Nature Materials* **2013,** *12* (11), 1038-1044.
17. Schmode, P.; Hochgesang, A.; Goel, M.; Meichsner, F.; Mohanraj, J.; Fried, M.; Thelakkat, M., A Solution-Processable Pristine PEDOT Exhibiting Excellent Conductivity, Charge Carrier Mobility, and Thermal Stability in the Doped State. *Macromolecular Chemistry and Physics* **2021,** *222* (20), 2100123.
18. Stöcker, T.; Köhler, A.; Moos, R., Why does the electrical conductivity in PEDOT:PSS decrease with PSS content? A study combining thermoelectric measurements with impedance spectroscopy. *Journal of Polymer Science Part B: Polymer Physics* **2012,** *50* (14), 976-983.
19. van de Ruit, K.; Cohen, R. I.; Bollen, D.; van Mol, T.; Yerushalmi-Rozen, R.; Janssen, R. A. J.; Kemerink, M., Quasi-One Dimensional in-Plane Conductivity in Filamentary Films of PEDOT:PSS. *Advanced Functional Materials* **2013,** *23* (46), 5778-5786.
20. Alshammari, B. A.; Al-Mubaddel, F. S.; Karim, M. R.; Hossain, M.; Al-Mutairi, A. S.; Wilkinson, A. N., Addition of Graphite Filler to Enhance Electrical, Morphological, Thermal, and Mechanical Properties in Poly (Ethylene Terephthalate): Experimental Characterization and Material Modeling. *Polymers* **2019,** *11* (9), 1411.
21. Choi, H.-J.; Kim, M. S.; Ahn, D.; Yeo, S. Y.; Lee, S., Electrical percolation threshold of carbon black in a polymer matrix and its application to antistatic fibre. *Scientific Reports* **2019,** *9* (1), 6338.
22. Brûlet, A.; Boué, F.; Cotton, J. P., About the Experimental Determination of the Persistence Length of Wormlike Chains of Polystyrene. *Journal de Physique II* **1996,** *6* (6), 885-891.

23. Kong, X.; Liu, J., An Atomistic Simulation Study on POC/PIM Mixed-Matrix Membranes for Gas Separation. *The Journal of Physical Chemistry C* **2019,** *123* (24), 15113-15121.
24. Zhang, F.; Mendelev, M. I.; Zhang, Y.; Wang, C.-Z.; Kramer, M. J.; Ho, K.-M., Effects of sub-Tg annealing on $Cu_{64.5}Zr_{35.5}$ glasses: A molecular dynamics study. *Applied Physics Letters* **2014,** *104* (6), 061905.
25. Zhang, Y.; Zhang, F.; Wang, C. Z.; Mendelev, M. I.; Kramer, M. J.; Ho, K. M., Cooling rates dependence of medium-range order development in $Cu_{64.5}Zr_{35.5}$ metallic glass. *Physical Review B* **2015,** *91* (6), 064105.
26. Eberly, D., Robust Computation of Distance Between Line Segments. **2020**.
27. Segal, D.; Nitzan, A.; Davis, W. B.; Wasielewski, M. R.; Ratner, M. A., Electron Transfer Rates in Bridged Molecular Systems 2. A Steady-State Analysis of Coherent Tunneling and Thermal Transitions. *The Journal of Physical Chemistry B* **2000,** *104* (16), 3817-3829.
28. Dijkstra, E. W., A note on two problems in connexion with graphs. *Numerische Mathematik* **1959,** *1* (1), 269-271.
29. Aryo, D., Dijkstra Algorithm. MATLAB Central File Exchange, 2021.
